# Recent Advances in Multilayer‐Structure Dielectrics for Energy Storage Application

**DOI:** 10.1002/advs.202102221

**Published:** 2021-09-14

**Authors:** Mengjia Feng, Yu Feng, Tiandong Zhang, Jinglei Li, Qingguo Chen, Qingguo Chi, Qingquan Lei

**Affiliations:** ^1^ Key Laboratory of Engineering Dielectrics and Its Application Ministry of Education Harbin University of Science and Technology Harbin 150080 P. R. China; ^2^ School of Electrical and Electronic Engineering Harbin University of Science and Technology Harbin 150080 P. R. China; ^3^ Electronic Materials Research Laboratory Key Lab of Education Ministry Xi'an Jiaotong University Xi'an 710049 P. R. China

**Keywords:** dielectric, energy storage density, multilayer‐structure dielectrics

## Abstract

An electrostatic capacitor has been widely used in many fields (such as high pulsed power technology, new energy vehicles, etc.) due to its ultrahigh discharge power density. Remarkable progress has been made over the past 10 years by doping ferroelectric ceramics into polymers because the dielectric constant is positively correlated with the energy storage density. However, this method often leads to an increase in dielectric loss and a decrease in energy storage efficiency. Therefore, the way of using a multilayer structure to improve the energy storage density of the dielectric has attracted the attention of researchers. Although research on energy storage properties using multilayer dielectric is just beginning, it shows the excellent effect and huge potential. In this review, the main physical mechanisms of polarization, breakdown and energy storage in multilayer structure dielectric are introduced, the theoretical simulation and experimental results are systematically summarized, and the preparation methods and design ideas of multilayer structure dielectrics are mainly described. This article covers not only an overview of the state‐of‐the‐art advances of multilayer structure energy storage dielectric but also the prospects that may open another window to tune the electrical performance of the electrostatic capacitor via designing a multilayer structure.

## Graphical abstract

In this review, the main physical mechanisms of polarization, breakdown, and energy storage in multilayer dielectric are introduced. The preparation methods and design ideas of multilayer dielectrics are mainly described. This article covers not only an overview of the state‐of‐the‐art advances of multilayer dielectric but also opens another window to tune the electrical performance of the electrostatic capacitor via designing a multilayer structure.

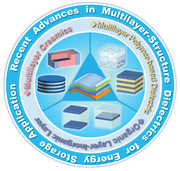

## 1 Introduction and Background

**Figure 1:**
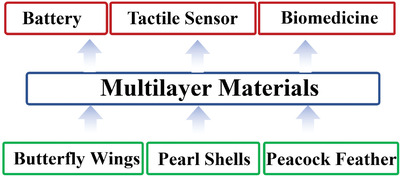
Multilayer materials in nature and applications of artificial multilayer materials.

In recent years, inspired by the multilayer structure in nature (e.g., abalone shells, peacock feathers, and butterfly wings), a variety of artificial multilayer structural materials have been successfully prepared and proven often have superior properties.^[^ ^1^ ^]^ As shown in **Figure** **1**, multilayer materials are widely used in various fields such as biomedicine,^[^ ^2^ ^]^ energy,^[^ ^3^ ^]^ tactile sensor,^[^ ^4^, ^5^ ^]^ metal materials,^[^ ^6^ ^]^ catalysis, power electronics,^[^ ^7^ ^]^ and signal system.^[^ ^8^ ^]^ Designing multilayer structural materials have become a promising method to improve the performance of dielectrics and has played a very important role in the exploitation of high energy‐storage performance dielectrics in particular.^[^ ^9^, ^10^, ^11^, ^12^ ^]^

Dielectric capacitors storage energy through a physical charge displacement mechanism and have ultrahigh discharge power density, which is not possible with other electrical energy storage devices (lithium‐ion batteries, electrochemical batteries or supercapacitors, and so on).^[^ ^13^, ^14^, ^15^, ^16^ ^]^ Dielectric capacitors are the key component of high frequency inverters, insulated‐gate bipolar transistor snubbers, pacemakers, defibrillators, high‐power lasers, and radars.^[^ ^17^ ^]^ Hence, dielectric materials for dielectric capacitors are of great research value.^[^ ^18^, ^19^ ^]^ It has attracted a lot of attention, and a series of exploratory work has been made to obtain high quality dielectrics for energy storage.^[^ ^20^, ^21^, ^22^, ^23^ ^]^ As researchers have explored more in the area of energy storage dielectrics, the understanding of how to design high‐performance energy storage dielectrics has deepened and related theories have been developed.^[^ ^23^, ^24^, ^25^ ^]^ At present, the energy storage density (*U*_e_) of biaxially oriented polypropylene (BOPP), the dielectric of commercial capacitors, is only 1–2 J cm^–3^.^[^ ^26^, ^27^, ^28^ ^]^ And in order to meet the need to store larger energy, dielectric capacitors are often designed to be larger in size and weight. Logically, the improvement of *U*_e_ is favorable to minimize the size and weight of dielectric capacitors and equipment largely and is advantageous for miniaturization and light weighting of equipment,^[^ ^29^ ^]^ such as mobile electronic devices,^[^ ^30^ ^]^ electromagnetic rail gun,^[^ ^15^, ^31^ ^]^ airplane,^[^ ^32^, ^33^ ^]^ and new energy vehicles.^[^ ^34^, ^35^, ^36^ ^]^ Energy storage density (*U*_e_) and efficiency (*η*) have been the most widely studied for they could directly assess the energy storage characteristics of materials used in dielectric capacitors.

**Figure 2:**
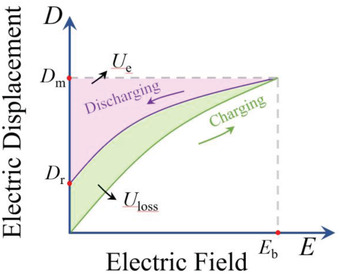
Calculation method of discharge energy storage density and loss.

As seen in **Figure** **2**, the calculation methodology for the discharge energy storage density is:^[^ ^37^, ^38^, ^39^, ^40^ ^]^

(1.1) $$U_{e}=\int_{D_{r}}^{D_{m}}E\;dD$$

where *E* and *D* are the electric field and electric displacement, *D*_r_ and *D*_m_ are the remnant and maximum electric displacement, respectively. It should be noted that since in many cases the electric displacement shift differs very little from the polarization (*P*), which is more readily available, many researchers have directly integrated the results calculated from the *P*–*E* loops as *U*_e_, as represented by^[^ ^41^ ^]^

(1.2) $$U_{e}=\int_{P_{r}}^{P_{m}}E\;dP$$

where *P* has a positive proportional relationship with the relative dielectric constant (*ε*_r_), therefore, it is important to enhance *ε*_r_ and *E*_b_ of the dielectric.^[^ ^37^, ^42^ ^]^

**Figure 3:**
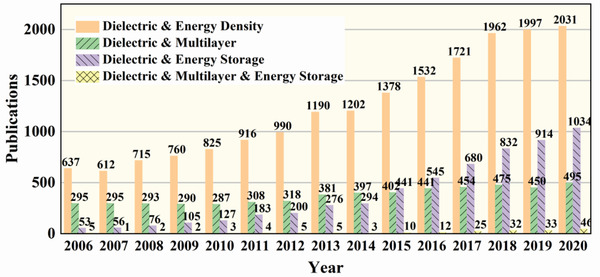
Trends in the number of articles on energy storage dielectrics published in the refereed journals from 2006 to 2020. The results were collected from Web of Science Core Collection using the keywords “dielectric” & “energy density,” “dielectric” & “multilayer,” “dielectric” & “energy storage,” “dielectric” & “multilayer” & “energy storage”, respectively.

In recent years, researchers used to enhance the energy storage performance of dielectrics mainly by increasing the dielectric constant.^[^ ^22^, ^43^ ^]^ As the research progressed, the bottleneck of this method was revealed.^[^ ^44^ ^]^ Due to the different surface energies, the nanoceramic particles are difficult to be evenly dispersed in the polymer matrix, which is a challenge for large‐scale production; in addition, the dielectric constant mismatch between the ceramic filler and the polymer matrix reduces *E*_b_ and reliability of composite dielectrics.^[^ ^45^, ^46^, ^47^, ^48^ ^]^ For ceramic‐based dielectric, the traditional ferroelectric ceramics have the problems of high loss and low breakdown. Usually, the new antiferroelectric has the problem of low polarization strength. It is difficult for one kind of dielectric to achieve the coexistence of high polarization and high breakdown. strength at the same time.^[^ ^11^ ^]^ The improvement of a certain polymer often requires a relatively large price and now it seems that combining different dielectric is an effective method. Among all the composite methods, the multilayer composite method has better controllability. Multilayer dielectric with strong controllability can be directed to combine the advantages of different materials and give full play to the role of the different dielectric. In addition, the interaction between ultrathin nanolayers will also bring interesting changes in certain characteristics of the dielectric.^[^ ^49^ ^]^ Therefore, the method is promised to facilitate the development of novel high‐performance dielectric for energy storage, and the simplicity of the multilayer structure helps to promote the development of related dielectric physics and chemistry disciplines. **Figure** **3** presents the trends in numbers of articles with the keyword “dielectric” & “energy density,” “dielectric” & “multilayer,” “dielectric” & “energy storage,” “dielectric” & “multilayer” & “energy storage” published in the refereed journals from 2006 to 2020. As can be seen from Figure 3, the number of publications concerning keywords above gradually increased from 2006 to 2020. In 2020, the highest number of articles with keywords “dielectric” & “energy density” was followed by articles with keywords “dielectric” & “energy storage,” then by articles with keywords “dielectric” & “multilayer,” and the lowest number of articles with keywords “dielectric” & “multilayer” & “energy storage.” In terms of percentage growth, from 2006 to 2020, the number of articles with “dielectric” & “energy density” as keywords increased by 218.8%, and the number of articles with the keywords “dielectric” & “multilayer” increased by 67.79%. The number of articles with the keywords “dielectric” & “energy storage” increased by 1850.9%, with articles with the keywords “dielectric” & “multilayer” & “energy storage” as keywords increased by 4500% (calculated using more representative data for 2007). The increasing number of published papers and the rapid growth rate indicates that the study of multilayer energy storage dielectrics has attracted great attention.

**Figure 4:**
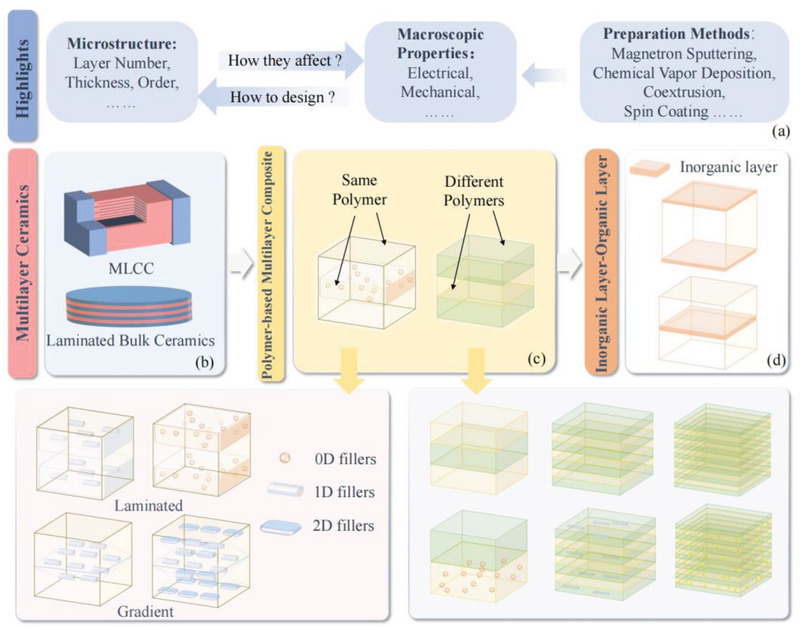
The focus of this review. a) Highlights. b) Multilayer ceramics. c) Polymer‐based multilayer composite. d) Inorganic–organic multilayer dielectric.

This review collects and organizes the work on multilayer structured energy storage dielectrics for the last 20 years. As shown in **Figure** **4**a, the influence of structural parameters (e.g., thickness, number of layers, interface density, layer order, layer pairing, etc.) on the relevant properties (breakdown, polarization, energy storage, lifetime) is described and summarized with emphasis. For ease of understanding, basic theories related to multilayer dielectric were first introduced in Chapter 2. The main focus is on the theory associated with the impact of multilayer microstructure on polarization properties and breakdown properties. Then, Chapter 3 summarizes the work on multilayer ceramic dielectric and multilayer polymer‐based dielectric. The line of thought is shown in Figure 4c–e. Ceramic capacitors are mostly used in miniature electronic products for bypass and filtering applications, which have low operating power and bear smaller voltage. Firstly, multilayer ceramic energy storage dielectrics are presented, including multilayer ceramic capacitors (MLCCs) and laminated ceramics films. The dielectric in MLCC is homogeneous, while structure of electrode is designed as multilayer; while the layered multilayer ceramic film has a dielectric consisting of more than two dielectric layers arranged in a certain order. Then, the research progress of thin film capacitors consisting of layered polymer materials is introduced. Polymer‐based capacitors have high resistance, are self‐healing and noninductive, can withstand high voltages, and are often used in pulsed power systems and inverter circuits. There is a wide variety of layered polymer‐based energy storage dielectrics, including those constructed by doping with inorganic nanofillers (Figure 4c), heterogeneous all‐organic multilayers (Figure 4d), doped heterogeneous multilayers (Figure 4e) etc. After a systematic introduction to their design ideas, Chapter 4 summarizes the processes and methods for the preparation of multilayer dielectrics. It should be noted that MLCCs are very widely used commercially and their state‐of‐the‐art preparation technology is on the production line rather than at the laboratory stage, so we will not go into details here but just make a brief introduction. Finally, a summary and challenges are given on the basis of the current state of development. Ceramic‐based energy storage dielectrics and polymer–polymer‐based energy storage dielectrics are comprehensively summarized and compared for the first time in this review, and the advantages and disadvantages of both dielectric materials are clearly presented. The preparation methods of both materials are summarized in detail. We elucidate the key factors for performing organic–inorganic bonding and give possible preparation methods. Relevant literature published from 2005 to the present is selected as the main reference for this review. This review will appeal to materials scientists, physicists, and chemists working in the dielectric physics community. We expect that this review will enable researchers to focus on the role of multilayer structures in enhancing the physical and electrical properties related to dielectrics and to improve the related theoretical system. How to design a multilayer structure so that the desired performance can be adjusted and enhanced should be regarded as an important issue.

## 2 Basic Theory of Multilayer Structure Dielectrics

Researchers have reached a consensus that the energy storage capacity of a material is inextricably linked to its dielectric and insulating properties. Achieving the synergistic elevation of polarization and dielectric strength has been the direction of researchers' efforts. Therefore, the polarization and breakdown models for multilayer dielectric are introduced systematically in this section.

### 2.1 Interface and Polarization in Multilayer Structure Dielectrics

The types and characteristics of polarization in multilayer dielectrics are introduced. Compared with a single homogeneous dielectric, one of the characteristics of a multilayer dielectric is that there is more interfacial polarization.^[^ ^50^, ^51^ ^]^ Therefore, the theory of interfacial polarization (i.e., Maxwell‐Sillar‐Wagner's model) is presented in detail.

#### 2.1.1 Maxwell‐Wagner Polarization

The Maxwell‐Wagner double‐layer dielectric model states that since the dielectric constants and conductivities of dielectrics are different, free charges (*σ*_f_) will accumulate at the interface.^[^ ^52^ ^]^

**Figure 5:**
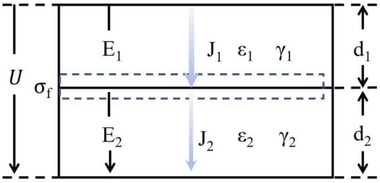
Double‐layer dielectric model.

As presented in **Figure** **5**, a step electric field of modulo U is applied to a double‐layer dielectric with no internal free charge. At the interface, according to Maxwell's equations:

(2.1) $$D_{1}=D_{2}\;$$

In the linear dielectric, there are:

(2.2) $$\varepsilon_{1}E_{1}=\varepsilon_{2}E_{2}$$

(2.3) $$U=E_{1}d_{1}+E_{2}d_{2}$$

Where *D*_1_ and *D*_2_ are the electric displacement of dielectric of materials 1 and 2, respectively. ɛ_1_ and ɛ_2_ stand the permittivity of material 1 and 2, respectively. *E*_1_ and *E*_2_ represent the electric strengths applied to material 1 and 2. The *d*_1_ and *d*_2_ denote the thickness of material 1 and 2, respectively.

According to Equations (2.2) and (2.3), the electric field strength can be obtained:

(2.4) $$\;E_{1}=\varepsilon_{2}U/\left(\varepsilon_{1}d_{2}+\varepsilon_{2}d_{1}\right)$$

(2.5) $$\;E_{2}=\varepsilon_{1}U/\left(\varepsilon_{1}d_{2}+\varepsilon_{2}d_{1}\right)$$

Current density (*J*) can also be obtained:

(2.6) $$J_{1}=\gamma_{1}E_{1}$$

(2.7) $$J_{2}=\gamma_{2}E_{2}$$

where *γ*_1_ and *γ*_2_ represent the conductivity of the two dielectric materials, respectively.

Since the current densities in the two materials are not equal (*J*_1_≠*J*_2_), the free charge accumulates at the interface, which in turn causes the electric field to change until the conduction currents are equal. The charge accumulation ends and the system reaches a stable state. So far, the charge accumulated at the interface (*σ*_f_) is the integral of current density over time.

(2.8) $$\sigma_{f}=\int [J_{1}-J_{2}]\;dt\;=\varepsilon_{0}\;\left(\gamma_{1}\varepsilon_{2}-\gamma_{2}\varepsilon_{1}\right)/\left(\gamma_{2}d_{1}+\gamma_{1}d_{2}\right)$$

According to the current continuum theorem:

(2.9) $$J\;=\gamma_{1}\;E_{1}+\varepsilon_{0}\varepsilon_{1}\;dE_{1}/dt=\gamma_{2}\;E_{2}+\varepsilon_{0}\varepsilon_{2}dE_{2}/dt$$

Set *τ*  = ɛ_0_(*d*_1_ɛ_2_ + *d*_2_ɛ_1_)/(*γ*_2_ *d*_1_ + *γ*_1_ *d*_2_)  as the time constant and solutions to equation are:

(2.10) $$\begin{array}{ccc} E_{1} & = & \gamma_{2}U/\left(\gamma_{2}d_{1}+\gamma_{1}d_{2}\right)+[\varepsilon_{2}/\left(d_{1}\varepsilon_{2}+d_{2}\varepsilon_{1}\right)-\gamma_{2}/(\gamma_{2}d_{1}\\ & & +\;\gamma_{1}d_{2})]Ue^{-\frac{1}{\tau }} \end{array}$$

(2.11) $$\begin{array}{ccc} E_{2} & = & \gamma_{1}U/\left(\gamma_{2}d_{1}+\gamma_{1}d_{2}\right)+[\varepsilon_{1}/\left(d_{1}\varepsilon_{2}+d_{2}\varepsilon_{1}\right)-\gamma_{1}/(\gamma_{2}d_{1}\\ & & +\;\gamma_{1}d_{2})]Ue^{-\frac{1}{\tau }} \end{array}$$

Equations (2.10) and (2.11) clearly show the time dependence of the electric field of the two dielectrics. Maxwell interface polarization describes the cumulative process of charge at the interface from transient to DC steady state, which has important theoretical significance.^[^ ^53^ ^]^ It should be pointed out that this theoretical model is relatively simple and only linear dielectric is considered. The theory of charge accumulation at the interface of nonlinear dielectric has not been clearly proposed.

Actually, Maxwell‐Sillar‐Wagner's model is so simple that it can hardly accurately describe the effect of multilayer structures on dielectric properties.^[^ ^54^ ^]^ Therefore, this review also summarizes other theoretical models related to the polarization properties of multilayer dielectric.

#### 2.1.2 Interfaces in Ceramic‐Based Dielectrics

**Figure 6:**
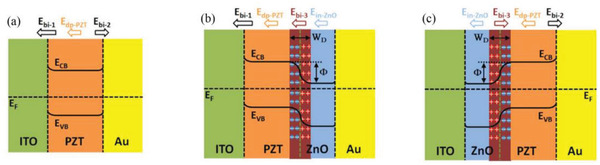
Energy bands and self‐built electric field distribution of heterojunctions. a) ITO/PZT/Au b) ITO/PZT/ZnO/Au c) ITO/ZnO/PZT/Au. Reproduced with permission.^[^ ^59^ ^]^ Copyright 2016, Springer Nature.

Two kinds of interfaces exist in energy storage ceramic dielectrics. One is the interface composed of materials with the same composition and structure but different polarization directions in the same material, called ferroelectric domain walls,^[^ ^55^ ^]^ which may have unique physical behavior.^[^ ^56^ ^]^ The other one refers to the interfaces between different materials, i.e., heterogeneous interfaces. Due to the presence of dangling bonds and incomplete atomic coordination, the polarity at interfaces is discontinuous.^[^ ^57^ ^]^ In addition, due to the differences in the energy band structures of different materials, carrier migration and energy band bending will occur when they come into contact with each other.^[^ ^58^ ^]^ A structure similar to a P–N junction is formed, as shown in **Figure** **6**. This special structure at the interface makes ferroelectric heterojunctions often exhibit specific electrical properties.^[^ ^59^, ^60^, ^61^ ^]^

When the multilayer structure is repetitive and the single layer is very thin, the superlattice phenomenon may occur. For example, it has been shown that a superlattice (KNbO_3_/KTaO_3_, PbTiO_3_/SrTiO_3_ and BaTiO_3_/SrTiO_3_) can change the Curie temperature by changing its period length, thus affecting other ferroelectric properties.^[^ ^49^, ^62^, ^63^, ^64^, ^65^, ^66^, ^67^ ^]^ In addition, the interaction of lattice, orbitals, charges, and spins at the heterogeneous interface produces peculiar physical properties, such as “improper ferroelectricity” in short‐period superlattices, ordered vortex arrays in mid‐period superlattices, and flux‐closed domain structures in large‐period superlattices.^[^ ^68^, ^69^, ^70^, ^71^, ^72^, ^73^ ^]^ These phenomena are important for researchers to understand the involved physical phenomena. The influence of this novel properties on the energy storage characteristics of multilayer dielectric needs to be explored.

#### 2.1.3 Interfaces in Polymer‐Based Dielectrics

##### Inorganic–Inorganic

Due to the excellent properties of filled composite dielectrics, there has been an increasing interest in inorganic particle‐polymer interfaces models, including Lewis's model^[^ ^74^ ^]^ and Tanaka's multinuclear model,^[^ ^75^ ^]^ etc. Excitingly, the interfacial polarization charge between BT and substrate was recently observed directly by AFM by Li et al.^[^ ^76^ ^]^ However, in multilayer structures, a filled dielectric with the same condition is considered as a macroscopic layer, which facilitates the simplification of the problem.

##### Inorganic Organic

Combining inorganic layers with organic layers to form a 2D–2D composite dielectric is also a novel and effective design‐side approach.^[^ ^77^ ^]^ Currently, mainly inorganic layers with high electron injection potential are coated on the polymer surface to increase the carrier injection potential.^[^ ^78^ ^]^

##### Organic–Organic

The analysis of organic–organic interfaces is more complicated. The interface causes changes in the molecular structure of the polymer, such as free volume fraction, mobility, crystallinity and conformation of polymer chains, which affect the dielectric properties of the polymer.^[^ ^79^, ^80^, ^81^, ^82^ ^]^ The analysis needs to be done according to the condition of the different polymers.

### 2.2 Breakdown Theory in Multilayer Dielectrics

#### 2.2.1 Electric Field Distribution in Multilayer Dielectrics

Calculating distribution of *E* in a composite dielectric is essential in predicting and improving *E*_b_. It is well known that the breakdown process of solids is very sophisticated, including electrical breakdown, thermal breakdown, and mechanical breakdown.^[^ ^83^, ^84^, ^85^, ^86^ ^]^ Obtaining the electric field distribution in complex structures through computational simulation and other methods, and designing for the weak points in them, is an important and effective means to improve the dielectric breakdown characteristics.^[^ ^87^, ^88^ ^]^ Therefore, the theory about distribution of electric fields in multilayer dielectric is presented here.

##### Electric Field Distribution in Linear Dielectrics

A linear dielectric is a dielectric that has a fixed dielectric constant when the electric field changes. Linear dielectrics typically have a relatively low *ε*_r_, low tan*δ* and a relatively high *E*_b_. In an ideal linear dielectric, the polarization strength grows linearly with the increasing *E* without hysteresis, i.e., as the electric field rises, the dielectric constant is basically constant.^[^ ^89^ ^]^ Therefore, the calculation of the distribution characteristics of the local *E* in the dielectric is slightly simpler: the partial voltage of the dielectric is related to the DC conductivity or relative dielectric constant of the dielectric.

Taking a double layer of dielectric as an example. When *E* is initially exerted on the dielectric, the electric displacement normal component is continuous at the interface, i.e., Equation (2.1). From Equation (2.2), it can be derived that in a composite dielectric made up of linear materials, the local electric field is antiproportional to the dielectric constant at that place. On this basis, the features of *E* distribution can be calculated as long as *ε*_r_ distribution in the composite dielectric is known. This calculation method is simple and easy to understand, although it is not considered comprehensively enough. Many studies have been based on this method for electric field distribution calculations.^[^ ^90^, ^91^ ^]^

##### Electric Field Distribution in a Nonlinear Dielectric

**Figure 7:**
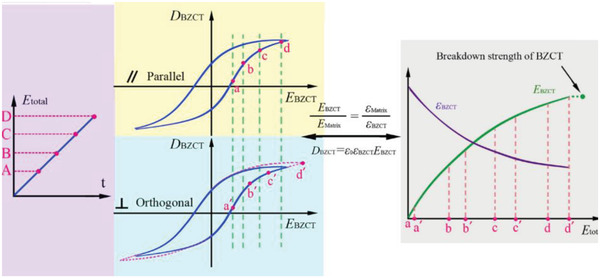
Electric field redistribution in PVDF‐based composite dielectrics with different structures. Reproduced with permission.^[^ ^48^ ^]^ Copyright 2019, Elsevier.

For ferroelectrics (ferroelectric ceramics, ferroelectric polymers or composite dielectric that exhibits ferroelectricity), the dielectric constant changes as the electric field increases, leading to a more complex distribution of the electric field for composite dielectric where nonlinear dielectrics are present. The peculiarities of the electric field distribution in nonlinear heterostructure dielectric have attracted the attention of researchers. For example, as shown in **Figure** **7**, Zhang et al. has discussed the dynamic invariance of the electric field distribution in polymer composites with nonlinear dielectric fillers.^[^ ^48^ ^]^

Under the high‐frequency electric field, there is not enough time for free charges to move and accumulate. According to Maxwell's equation, the electric displacement *D* is continuous at the interface, i.e., formula (2.1),

(2.12) $$D=\varepsilon_{0}E+P$$

(2.13) $$\varepsilon_{0}E_{1}+P_{1}=\varepsilon_{0}E_{2}+P_{2}$$

**Figure 8:**
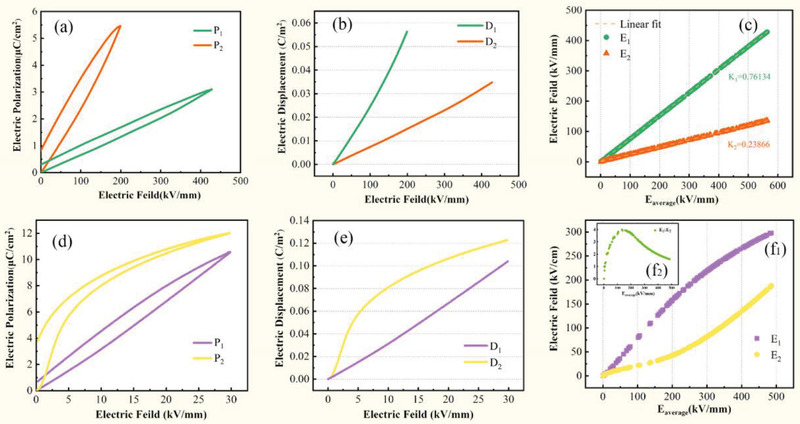
Method of calculating the voltage divided by each layer for a multilayer dielectric. a) *P*–*E* Loops, b) Calculated *D*–*E* relationships and c) Electric field distribution in linear multilayer composite dielectric. d) *P*–*E* loops, e) Calculated *D*–*E* relationships and f_1_) Electric field distribution in non‐linear multilayer composite dielectric, f_2_) The variation of *E*_1_:*E*_2_ with *E*_average_ change.

According to formula (2.13) and the *P*–*E* loops of the material, the electric field distribution in the nonlinear composite dielectric can be calculated. In this way, the electric field distributions in linear‐linear double‐layer heterodiegetic and linear‐nonlinear double‐layer heterodiegetic are calculated. **Figure** **8**a,c are the *P*‐*E* loops of the linear–linear double‐layer composite dielectric and the linear‐nonlinear double‐layer composite dielectric, respectively. Convert *P* to *D* according to formula (2.12) and the obtained *D*–*E* relationships are shown in Figure 8b,e. According to Figure 8b,e, the relationships between the partial electric field (*E*_1_ and *E*_2_) and the total electric field (*E*) are plotted in Figure 8c,f_1_. Comparing the two figures, it can be found that the existence of nonlinear dielectric indeed affects the electric field distribution. This also proves that considering the characteristics of nonlinear material is of great significance for accurately solving the electric field distribution in heterostructure dielectric. In Figure 8f_2_, the ratio of the partial electric fields *E*_1_ and *E*_2_ (i.e., *E*_1_:*E*_2_) is plotted as the function of the total electric field strength (*E*). As seen, *E*_1_ *:E*_2_ is not constant but increases and then decreases as the electric field becomes larger. In other words, the appearance of the maximum local electric field value is not necessarily at the end of the process of applying an increasing electric field. This reflects the significance of studying intermediate processes.

##### The Impact of the Applied Voltage Waveform

From Equations (2.10) and (2.11), it can be seen that both *E*_1_ and *E*_2_ are related to time (*t*). When applied voltage is AC, the period (*T*) determines the values of *E*_1_ and *E*_2_. And when *T* is much larger than 3*τ*‐5*τ*, or when a DC, step voltage is applied, the conductivity of two dielectric determines the electric field distribution. To be precise, not only the frequency but also the time and waveform of the applied voltage affect the electric field distribution, which is worth further exploration.

#### 2.2.2 Occurrence of Breakdown in Multilayer Dielectrics

##### Increase in Breakdown Path along the Interface Direction

Due to the different local electric fields in different layers of the multilayer dielectric and the presence of interfaces, the breakdown occurs with unique characteristics. Encouragingly, the interface in the multilayer structure was shown to have the effect of blocking the development of electric dendrites.^[^ ^92^, ^93^, ^94^, ^95^, ^96^ ^]^

**Figure 9:**
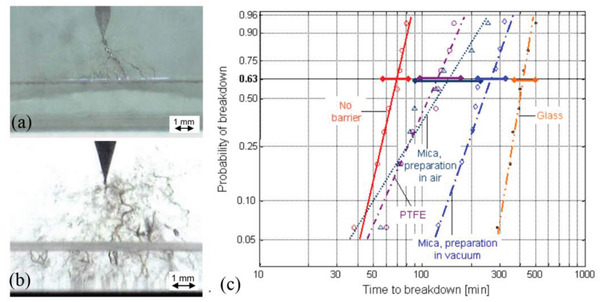
Tree figures at a barrier that can: a) not be penetrated by the tree (0.2 mm mica) or b) easily be penetrated by the tree (0.01 mm PETP‐film). c) Weibull‐plot for the time to breakdown values for tests of samples with no barrier and barriers at different interfacial strength. Reproduced with permission.^[^ ^96^ ^]^ Copyright 2006, IEEE.

The blocking effect of the interface on the electric tree has been widely observed. As shown in **Figure** **9**a, the length of electric tree could be prolonged by the barrier layer and the time of breakdown process could also be increased. And when the blocking layer is too thin, it will not work (see Figure 9b). The properties of the barrier layer (e.g., hydrophobicity) can also affect the effect. For example, glass and mica surfaces are hydrophilic, resulting in relatively strong van der Waals forces on the substrate. In contrast, PTFE has no or only weak van der Waals forces and is therefore less effective in blocking. What can be discerned from Figure 9c is that the dielectric with PTFE barriers present a shorter time to breakdown.

**Figure 10:**
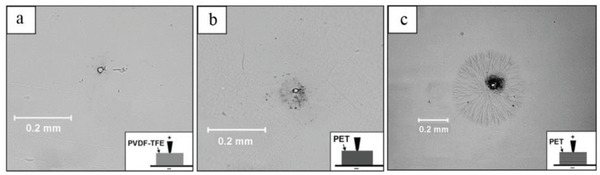
Fracture damage zones of a single layer of a) extruded P(VDF‐TFE), b) extruded PET film, c) extruded multilayer film in the electric tree. Reproduced with permission.^[^ ^97^ ^]^ Copyright 2013, Wiley‐VCH.

The states of the electric tree in monolayer and in a multilayer dielectric are shown in **Figure** **10**. All films were fractured at a constant electric field of 1200 kV mm^–1^ using needle/plane electrodes.^[^ ^97^ ^]^ The results show that the damage zones of the multilayered samples were substantially different when compared with the single‐layer controls. All multilayered samples exhibit distinct treeing patterns surrounding a single breakdown hole. The single‐layer controls fractured at 1200 kV mm^–1^ possessed only a single breakdown hole. And it shows the rule that the larger the breakdown strength, the larger the electric tree diameter. This study confirms the phenomenon of lateral growth of electric tree branches at the interface between layers, i.e., the interface vertical to applied voltage can significantly diminish the growth of electric trees along the direction of electric field.

##### Simulation of Breakdown Process

**Figure 11:**
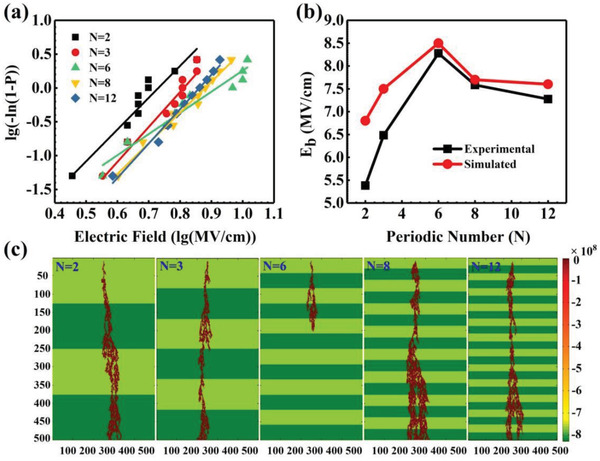
a) Comparison of breakdown strength of dielectrics with varied layer number and b) experimental breakdown strength results compared with simulation results. c) Development of the breakdown path in a multilayer system, simulated under *E* of 8 MV cm^–1^. Reproduced with permission.^[^ ^98^ ^]^ Copyright 2018, Elsevier.

Simulation is an important research tool, with the help of which human and material resources can be saved. Currently, there are two main types of simulations of breakdown paths in multilayer dielectric: one based on the dielectric breakdown model (DBM)^[^ ^99^, ^100^ ^]^ and the other based on the phase field method.^[^ ^101^, ^102^, ^103^ ^]^ As shown in the **Figure** **11**, breakdown paths in multilayer dielectric can be simulated. The breakdown paths in the multilayer were simulated and confirmed to be in good agreement with the actual measurement results.^[^ ^98^ ^]^ What can be concluded from the simulations result is that the appropriate number of layers and thickness help to block the breakdown path.

**Figure 12:**
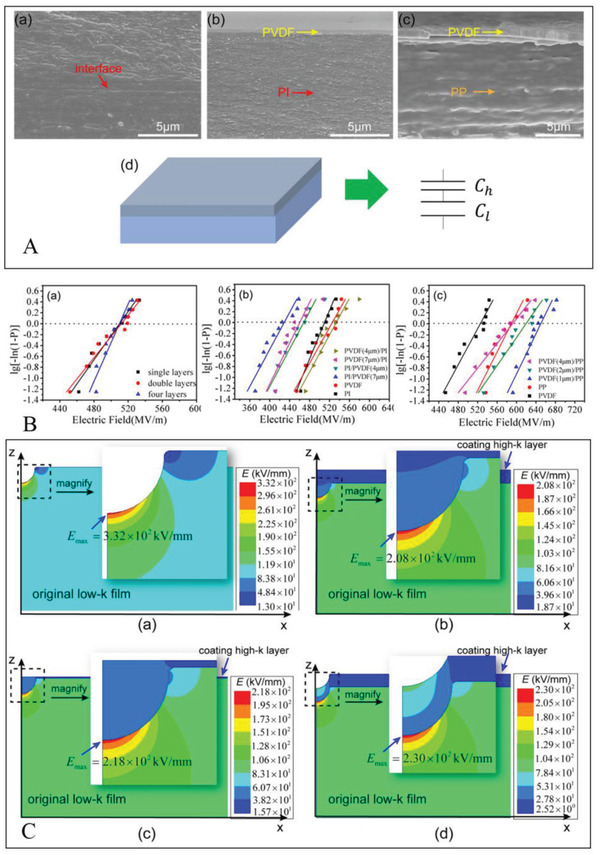
A‐a–c) Cross‐sectional SEM images of double‐layer dielectric composed of diverse materials. d) A diagram of the double‐layer dielectric and the corresponding series capacitor model. B) *E*_b_ of different bilayer dielectrics. C) Simulation of *E* distribution in dielectric with the same size defects coated with high dielectric layers of different thicknesses. Reproduced with permission.^[^ ^104^ ^]^ Copyright 2019, AIP Publishing.

Dang et al. have examined the impact of factors such as multilayer location on the breakdown performance of composite dielectrics and its mechanism.^[^ ^104^ ^]^ Several bilayers composed of different types of polymers were successfully prepared and used to explore the mechanism of enhanced breakdown performance. As shown in **Figure** **12**A, both bilayers composed of the same material (PP/PP) and bilayers composed of different materials (PVDF/PI and PVDF/PP) are included. The results show that the interface between layers of the same material may have virtually no impact on its *E*_b_. Secondly, *E*_b_ changes when the two surfaces of the same sample are switched with fixed polarity of the applied DC voltage. That is, changing the relative position of the layer to the electrode in the bilayer film does have some effect on *E*_b_. And when the layer with higher *ε*_r_ is in contact with the negative electrode, the thinner the higher dielectric constant layer is, the more significant the breakdown strength enhancement effect of the multilayer material is. This can be attributed to the fact that materials with different dielectric constants have different electron injection when they are in contact with the electrode. Finally, the simulation results show that the high polarization layer of the coating can effectively suppress the local *E* distortion around the defect, as shown in Figure 12B.

##### Carrier Injection Barrier

**Figure 13:**
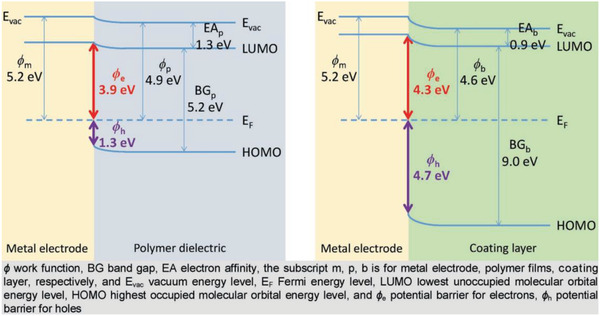
Schematic diagram of the variation of the carrier injection potential for different materials_._ Reproduced with permission.^[^ ^78^ ^]^ Copyright 2018, Wiley‐VCH.

It is well known that carrier injection at the electrode is one of the main reasons for breakdown (especially at high temperatures).^[^ ^44^ ^]^ Therefore, the carrier injection potential at the electrode needs to be attended to. And in a multilayer structured dielectric, the carrier injection barrier can be increased by structural design. As shown in **Figure** **13** diagram, the injection potential of electrons is raised by changing the dielectric in contact with the electrode.

## 3 Superiority of Multilayer Structure dielectric for Energy Storage

### 3.1 Multilayer Ceramic Energy Storage Dielectrics

Ceramics are suitable for dielectric capacitors due to their ultrahigh dielectric constant (up to thousands for ferroelectric).^[^ ^105^, ^106^, ^107^, ^108^, ^109^, ^110^ ^]^ But ceramic dielectrics also have a high dielectric loss, relatively low breakdown strength and other problems that need to be solved. Many studies have been conducted on ceramic dielectric in order to achieve reinforced energy storage capability.^[^ ^111^ ^]^

#### 3.1.1 Multilayer Ceramic Capacitors (MLCCs)

**Figure 14:**
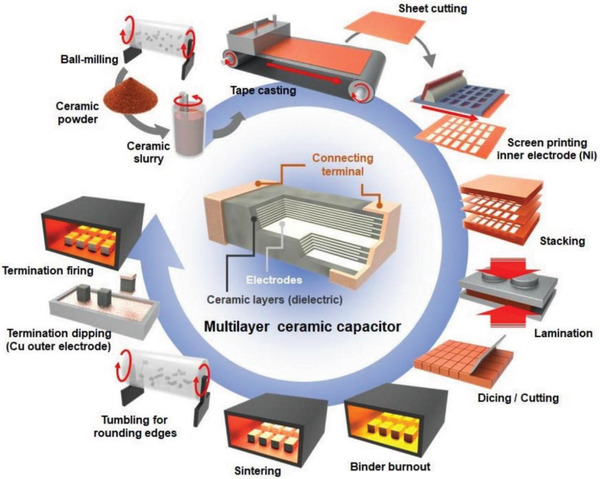
Schematics of MLCC architecture and its fabrication process. Reproduced with permission. ^[^ ^118^ ^]^ Copyright 2019, Royal Society of Chemistry.

Ceramic capacitors have been used for energy storage purposes for more than 60 years, which has a vital role in the field of power electronics and pulsed power systems due to their small footprint, excellent temperature stability (up to 250 °C) and high *ε*_r_.^[^ ^35^, ^89^, ^112^, ^113^, ^114^, ^115^, ^116^ ^]^ It is well known that the capacity of a capacitor is inversely proportional to the distance between the electrodes and directly proportional to the area of the electrodes. Therefore, those ceramic dielectrics that are made into a multilayer structure can obtain a larger capacitance capacity. Multilayer ceramic capacitors (MLCCs) are the main form of ceramic capacitors due to their high capacity and compactness,^[^ ^7^ ^]^ and have very wide applications such as resonant, buffer, by‐pass and coupling in mobile phone, motor vehicles and laptops.^[^ ^117^ ^]^ The structure and fabrication procedure of MLCC is shown in **Figure** **14**. The capacitance (*C*) of the MLCCs can be expressed as *C* = *ε*_r_ *ε*_0_(*n* − 1)*A*/*d*, where *ε*_r_ is the relative dielectric permittivity, *ε*_0_ is the vacuum permittivity, *n* is the number of the stacked inner electrode, *A* is the overlapped area of internal electrode and *d* is the thickness of the dielectric layer.^[^ ^118^ ^]^ The main approaches to develop next‐generation MLCCs are as follows. Firstly, the thickness of the dielectric monolayer *d* can be reduced.^[^ ^117^ ^]^ Secondly, *ε*_r_ can be increased by using energy storage dielectric materials with better performance,^[^ ^119^ ^]^ and the reliability of the capacitor can be improved by using electrode materials with better performance. In addition to these, increasing the overlap area *A* of the internal electrodes is also effective. Finally, improving the co‐sintering process is also a valid and major way to improve the reliability of capacitors.

In technical aspect, many companies have always been committed to reducing dielectric thickness. In order to maintain a smooth dielectric surface, the size of the powder material needs to be controlled below 300 nm.^[^ ^117^ ^]^ Control the sintering atmosphere and the sintering temperature are also important because high sintering temperature (above 1200°C) may cause the metal diffusion into the dielectric layer and high oxygen pressure may cause the oxidation of the base metal inner electrode. For example, the improvement methods of electrode structure and sintering rate were investigated by Cai et al.^[^ ^120^, ^121^ ^]^

For dielectric materials, the energy storage characteristics of different material MLCCs are summarized in **Table** **1**. Recent studies have shown that antiferroelectric (AFE) and relaxor ferroelectric (RFE) materials have great potential to improve the energy storage characteristics of MLCC. For example, Li et al. prepared (Na_0.5_Bi_0.5_)TiO_3_‐0.45(Sr_0.7_Bi_0.2_)TiO_3_ multilayer ceramic capacitors by combining AFE and RFE, and achieved an energy storage density of 9.5 J cm^–3^ and an ultra‐high energy storage efficiency of 92%.^[^ ^36^ ^]^ Wang et al. utilize stoichiometric doping reduced the electrical conductivity and electrical homogeneity and achieved an energy storage density of ≈13.8 J cm^–3^ in a 0.57BF‐0.30BT‐0.13BLN MLCC.^[^ ^122^ ^]^ The effect of doped Nd on the energy storage performance of BF‐based ceramics was systematically investigated by Wang et al.^[^ ^123^ ^]^ In addition, to address the problem of ceramic capacitors with high strain and prone to breakdown failure, Li et al. improved Weibull breakdown strength by preparing high‐quality <111>‐textured Na_0.5_Bi_0.5_TiO_3_–Sr_0.7_Bi_0.2_TiO_3_ with a lower strain induced by the electric field.^[^ ^106^ ^]^

**Table 1:** MLCCs and its related electrical properties

Materials	Thickness	Internal electrode	*E*_b_ [kV mm^–1^]	*W*_rec_ [J cm^−3^]	*η* [%]	Refs.
0.7BaTiO_3_–0.3BiScO_3_	25	Pt	73	6.1	–	^[^ ^124^ ^]^
Ca(Zr,Ti)O_3_	10	Pt	150	4	–	^[^ ^125^ ^]^
(Pb_0.88_Ba_0.05_La_0.02_Dy_0.04_)(Zr_0.68_Sn_0.27_Ti_0.05_)O_3_	20	Pd/Ag (95/5)	30	2.7	70	^[^ ^126^ ^]^
[0.94(0.75NBT−0.25NN)−0.06BT]−0.1CaZrO_3_	30	Ag/Pd (70/30)	12	0.34	88	^[^ ^127^ ^]^
BaTiO_3_−0.12Bi(Li_0.5_Ta_0.5_)O_3_	30	Pt	28	4.05	95	^[^ ^128^ ^]^
0.75(Bi_0.75_Nd_0.15_)FeO_3_‐0.25BaTiO_3_‐0.1wtMnO_2_	32	Pt	54	6.74	77	^[^ ^123^ ^]^
BaTiO_3_−0.13Bi[Zn_2/3_(Nb_0.85_Ta_0.15_)_1/3_]O_3_	11	Ag/Pd (60/40)	79	7.8	88	^[^ ^120^ ^]^
BaTiO_3_−0.13Bi[Zn_2/3_(Nb_0.85_Ta_0.15_)_1/3_]O_3_	11	Ag/Pd (60/40)	75	8.13	95	^[^ ^121^ ^]^
BaTiO_3_‐(Bi_0.5_Na_0.5_)TiO_3_	30	Ag/Pd	45	2.76	84	^[^ ^129^ ^]^
BaTiO_3_−0.12Bi(Li_0.5_Nb_0.5_)O_3_	29	Pt	45	4.5	91	^[^ ^16^ ^]^
BiFeO_3_−0.3 BaTiO_3_−0.08Nd(Zr_0.5_Zn_0.5_)O_3_	16	Pt	70	10.5	87	^[^ ^130^ ^]^
0.87BaTiO_3_‐0.13Bi(Zn_2/3_(Nb_0.85_Ta_0.15_)_1/3_)O_3_	5	Ag/Pd	104	10.12	89	^[^ ^131^ ^]^
<111>‐textured Na_0.5_Bi_0.5_TiO_3_–Sr_0.7_Bi_0.2_TiO_3_	20	Pt	103	21.5	82	^[^ ^106^ ^]^
0.5BiFeO_3_‐0.4SrTiO_3_‐0.03Nb‐0.1BiMg_2/3_Nb_1/3_O_3_	8	Pt	100	15.8	75	^[^ ^132^ ^]^
BNSr_0.4_TNb_2.5_Cu_0.8_Mn_0.15_	27	Ag/Pd (75/25)	27	2.83	85	^[^ ^133^ ^]^
0.61BiFeO_3_−0.33(Ba_0.8_Sr_0.2_)TiO_3_−0.06La(Mg_2/3_Nb_1/3_)O_3_	7	Pt	73	10	72	^[^ ^134^ ^]^
0.57BiFeO_3_−0.3BaTiO_3_−0.13Bi(Li_0.5_Nb_0.5_)O_3_	8	Pt	40	13.8	81	^[^ ^122^ ^]^
BaTiO_3_−0.13Bi[Zn_2/3_(Nb_0.85_Ta_0.15_)_1/3_]O_3_	5	Ag/Pd (60/40)	104	10.1	89	^[^ ^135^ ^]^
BaTiO_3_−0.13Bi[Zn_2/3_(Nb_0.85_Ta_0.15_)_1/3_]O_3_	9	Ag/Pd (60/40)	100	10.5	93	^[^ ^135^ ^]^
BaTiO_3_−0.13Bi[Zn_2/3_(Nb_0.85_Ta_0.15_)_1/3_]O_3_	11	Ag/Pd (60/40)	76.5	7.81	97	^[^ ^135^ ^]^
BaTiO_3_−0.13Bi[Zn_2/3_(Nb_0.85_Ta_0.15_)_1/3_]O_3_	20	Ag/Pd (60/40)	57.8	5.64	98	^[^ ^135^ ^]^
BaTiO_3_−0.13Bi[Zn_2/3_(Nb_0.85_Ta_0.15_)_1/3_]O_3_	26	Ag/Pd (60/40)	51.1	4.89	95	^[^ ^135^ ^]^
0.62Na_0.5_Bi_0.5_TiO_3_‐0.3Sr_0.7_Bi_0.2_TiO_3_‐0.08BiMg_2/3_Nb_1/3_O_3_	8	Pt	100	18	93	^[^ ^136^ ^]^

#### 3.1.2 Multilayer Ceramic Films

Besides the increase of dielectric energy storage density, the main problem of multilayer ceramic films is the large residual polarization leading to low efficiency and low breakdown strength. Therefore, combining ferroelectric and paraelectric materials or using relaxed ferroelectric, antiferroelectric materials are better choices than using ferroelectric ceramics alone. Compared with single‐layer dielectric, after combining two and more ceramic dielectric phases in a layered structure, there are some special effects in the dielectric, such as heterojunction effect,^[^ ^137^ ^]^ interfacial “dead‐layer,”^[^ ^138^ ^]^ space‐charges effect,^[^ ^63^ ^]^ electric field redistribution,^[^ ^91^ ^]^ etc. When designing multilayer ceramic films, these unique effects should be fully utilized.^[^ ^139^ ^]^

##### Ferroelectric/Paraelectric

Ferroelectrics can be spontaneously polarized in a certain temperature range, which are always used as energy storage dielectric for the large maximum polarization. However, most ferroelectric materials have the problem of large residual polarization, which result in a lower energy storage density and generates heat to reduce the service life of the equipment. Linear ceramics have higher breakdown strength and lower dissipation, but a lower *ε*_r_. In short, neither of them alone is satisfactory as an energy storage material. Combining ferroelectric/paraelectric ceramics with different and complementary properties to construct multilayer structured energy storage dielectrics is a unique and efficacious way for improving energy storage capacity. Compared to polymer‐based multilayer structured dielectrics, multilayer ceramic dielectrics are unique in their superlattice, dead‐layer effects^[^ ^62^ ^]^ and therefore deserve to be explored in greater depth.

**Figure 15:**
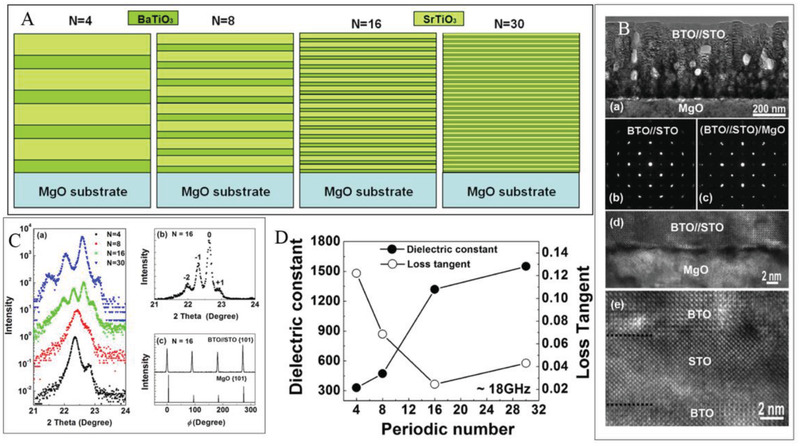
A) Sketch, B) cross‐sectional TEM studies, and C) typical XRD pattern of the as‐grown BT/ST multilayered films. D) Periodic number dependent on the value of *ε*_r_ and tan*δ* of the as‐grown BT/ST multilayered films. Reproduced with permission. ^[^ ^65^ ^]^ Copyright 2012, American Chemical Society.

As described in previous section, the dielectric behavior of multilayer dielectrics can be tuned by controlling the size‐parameters (e.g., the thickness of each layer in a multilayer ceramic). It is always known that the energy storage properties of the dielectric are closely related to the dielectric properties. Therefore, adjusting the parameters of the superlattice structure is an effective method for its energy storage modulating properties. For example, a representative and widely studied combination is BaTiO_3_ and SrTiO_3_.^[^ ^49^, ^64^, ^140^, ^141^, ^142^ ^]^ As shown in **Figure** **15**A, BaTiO_3_/SrTiO_3_ multilayer films were prepared on single‐crystal MgO substrates using an excimer pulsed laser deposition system. Number of superimposed periods for BaTiO_3_ and SrTiO_3_ monolayers were 4, 8, 16, and 30, respectively, and the layer thicknesses were 6 and 9 nm for SrTiO_3_ and BaTiO_3_, respectively, for a total film thickness close to 450 nm. All these samples were designed to control the same film thickness with equal total deposition time. As the periodic number increases or the thickness decreases, the dielectric constant increases while the tangential of dielectric loss rapid decrease, which theoretically will increase the energy storage density of dielectric.^[^ ^143^ ^]^

There are quite a lot of research on ferroelectric‐paraelectric superlattices, but more attention has been paid to their dielectric properties and less has been paid to their energy storage properties. More relevant studies are expected to emerge.

##### Relaxed Ferroelectrics

In contrast to ferroelectrics, relaxed ferroelectrics (e.g., PbMg_1/3_Nb_2/3_O_3_, PbZn_1/3_Nb_2/3_O_3_, PbSc_1/2_Nb_1/2_O_3_, SrTiO_3_–Na_0.5_Bi_0.5_TiO_3_, and BaTiO_3_–BiZn_0.5_Ti_0.5_O_3_^[^ ^144^ ^]^) lack a long‐range ordered structure and are considered to be bipolar glasses. It owns nearly ignorable remanent polarization and coercive electric field, and also shows more pronounced ferroelectric hysteresis. Therefore, it is preferred from a material point of view and becomes a suitable choice for materials used in capacitors.^[^ ^145^, ^146^ ^]^ Therefore, we outline the work using relaxed ferroelectric materials as multilayer dielectric elements.

**Figure 16:**
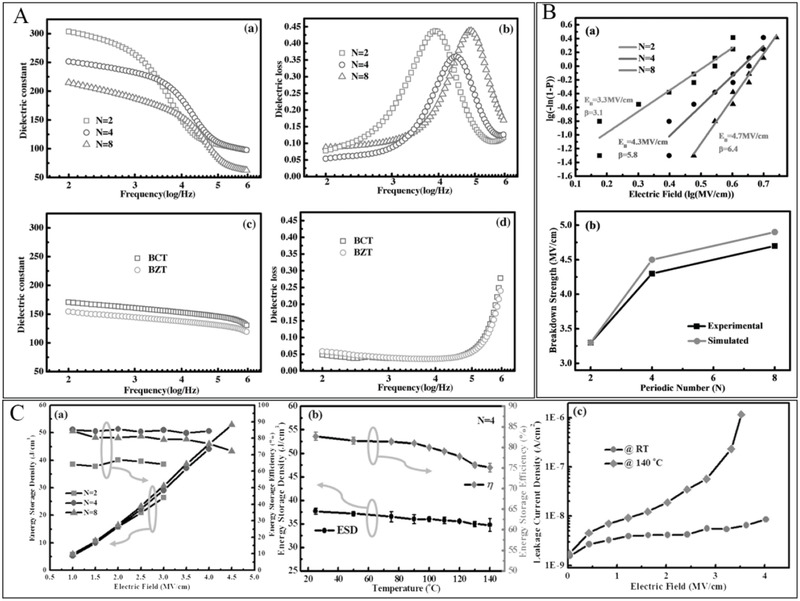
A) Plots of dielectric properties of multilayer and monolayer dielectrics. B) Experimental measured and simulated values of multilayer dielectric breakdown performance. C) Energy storage property and temperature stability of *U*_e_, *η*, and leakage current of multilayer dielectric. Reproduced with permission. ^[^ ^147^ ^]^ Copyright 2017, Wiley‐VCH.

Pb‐free composite ceramic, Ba_0.7_Ca_0.3_TiO_3_–BaZr_0.2_Ti_0.8_O_3_ (BCT‐BZT), is considered the most promising alternative to environmentally unfriendly lead‐based ceramics. It exhibits a very high dielectric constant attributed to coexistence at the metastable phase boundary.^[^ ^148^ ^]^ Multilayer structural ceramics consisting of BCT‐BZT also exhibit excellent energy storage properties. As shown in **Figure** **16**A, the dielectric properties of BCT/BZT multilayer ceramics are very different from those of single‐layer dielectrics. All multilayer structured ceramics have high dielectric constants and exhibit relatively large dispersion. The high *ε*_r_ was attributed to the contribution of interface polarization. The dielectric loss‐frequency function changes as periodic number (N) varies. Ceramics with different cycle numbers exhibit different relaxation peaks. From Figure 16B, it is observed that the number of periods has a positive relationship with the breakdown strength of the ceramic. Combined with the simulation analysis, the interface may be a factor blocking the development of the breakdown path, and *E*_b_ increases as the number of interfaces increases. As shown in Figure 16C, through engineering interfaces, both the dielectric and insulation properties of the dielectric can be effectively improved, leading to the energy storage performance enhancement. The results proved that the energy storage density (*U*_e_) of the dielectric with layer number 8 reached more than 50 J cm^–3^ and the efficiency reached more than 70% at room temperature. The experimental data also show that the multilayer structure exhibits excellent temperature stability. Although researchers have conducted a series of surveys, the impacts of thickness on dielectric behavior are yet unknown.^[^ ^147^ ^]^

**Figure 17:**
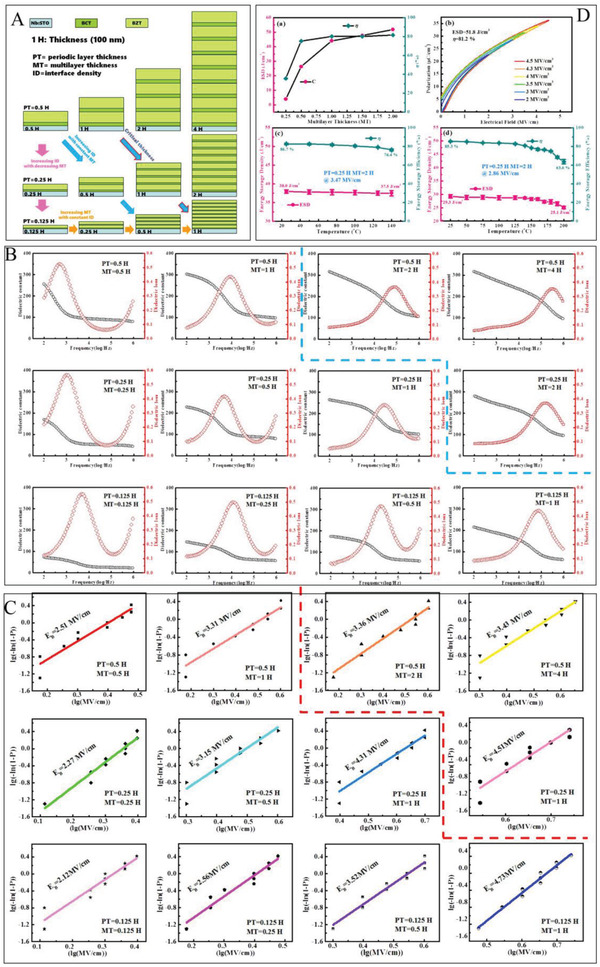
A) Sketch for the BCT/BZT multilayer films. B) Dielectric and C) breakdown properties for all the BCT/BZT multilayer films. D) The performances of the multilayer films with PT = 0.25 H at RT and high temperature. Reproduced with permission.^[^ ^149^ ^]^ Copyright 2018, The Royal Society of Chemistry.

The study of interface engineering is somewhat complex, mainly due to the complexity of the control variables. The total thickness, interfaces number, and the thickness of the monolayer are variables that can affect the energy storage characteristics of the dielectric. To streamline the thinking, the concept of interface density can be introduced. As shown in **Figure** **17**A, only the total thickness (Pink arrow direction) and the interfacial density (Yellow arrow direction) need to be varied to perform the study. The dielectric properties in the frequency scope of 100 Hz‐1M Hz and breakdown performance corresponding to Figure 17A are shown in Figure 17B,C, respectively. As seen, Figure 17B,C are both divided into two parts by a dashed line. In the lower left section of Figure 17B, at a fixed cycle thickness, adding the total thickness of the multilayer film is concomitant with an increase in the dielectric constant and a decrease in the dielectric, which is regarded as the impact of a “dead layer.” The forming of dead layers may be attributed to a mismatch between the substrate and the film or between the film and the electrode, as well as due to stress.^[^ ^150^, ^151^ ^]^ The dead layer effects can be ignored when the total thickness exceeds 100 nm. Therefore, in the upper right part, the variation pattern is different from that of the lower left part. At the equal interface number, *ε*_r_ increases monotonically with the increase of the period thickness, which has been attributed to the increase in interface polarization. However, tan*δ* exhibits the opposite tendency. The reduction in dielectric dissipation can be attributed to the reduction in crystallinity. As shown in Figure 17C, when the number of interfaces is stationary, *E*_b_ of the multilayer dielectric increases with the increase of the one‐cycle thickness, while the right section shows the contrary trend. When the overall thickness of the multilayer is lower than the critical value, i.e., ≈100 nm as previously discussed, the breakdown strength drops with the increase of the single‐cycle thickness (i.e., the decrease of the interface density), as has been reported in previous work.^[^ ^147^ ^]^ In this multilayer system, the dielectric constant can reach a maximum of ≈400, and the breakdown strength can reach 472 MV m^–1^. As shown in Figure 17D, *U*_e_ of multilayer ceramics is relatively outstanding at both room and high temperature (200 °C)

**Figure 18:**
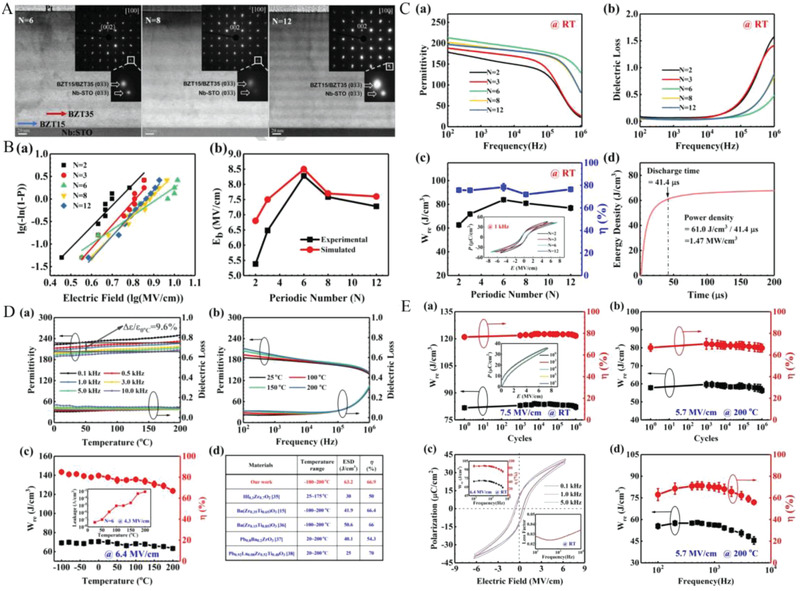
A) STEM images of multilayer film cross‐sections and selected area electron diffraction (SAED) patterns. B) Experimental and simulation results of the electric breakdown strength of multilayer dielectrics. C–E) Dielectric, energy storage, and fatigue properties at room temperature and high temperature. Reproduced with permission.^[^ ^98^ ^]^ Copyright 2018, Elsevier.

Multilayer ceramics consisting of BZT also exhibit remarkable energy storage properties especially in the wide temperature range,^[^ ^19^ ^]^ because the single‐phase BaZr_0.35_Ti_0.65_O_3_ (BZT35) exhibited excellent high‐temperature energy storage characteristics.^[^ ^152^ ^]^ As shown in **Figure** **18**A, BZT15 and BZT35 have close lattice constants, which could greatly diminish the dislocations across the interface induced by lattice mismatch. Thus, *E*_b_ can be increased. The Weibull distribution of breakdown strength has been shown in Figure 18B, and the breakdown intensity obtained is summarized and compared with the simulation results (red squares). With a stationary overall dielectric thickness, the breakdown strength initially grows with the number of cycles, hits a maximal value at *N* = 6, and then drops marginally. It can be inferred that interfaces in multilayer dielectrics can indeed help resist the progression of breakdown channels penetrating the film. On the other hand, as increasing the number of interfaces (reduction in the thickness of each layer), the interfacial coupling is getting stronger and the interfacial blocking effect is weakened. In addition, interfacial diffusion is probably another major contributor to weakening the interfacial barrier effect. In the corresponding magnified image of Figure 18A, it can be noticed that the interface of the dielectric becomes rough for more layers. Seven‐layer multilayer ceramics exhibit the finest breakdown strength and the optimum dielectric properties, i.e., the largest *ε*_r_ and the minimum tan*δ*. The inclusion of BZT15 is expected to improve the high temperature energy storage characteristics of the composite dielectric because of its higher dielectric operating temperature compared to BZT35. The BZT15/BZT35 multilayer film has indeed proven to exhibit good temperature stability, stable high fatigue endurance over a long period of time, and a wide range of operating frequencies (as shown in Figure 18D,E).

**Figure 19:**
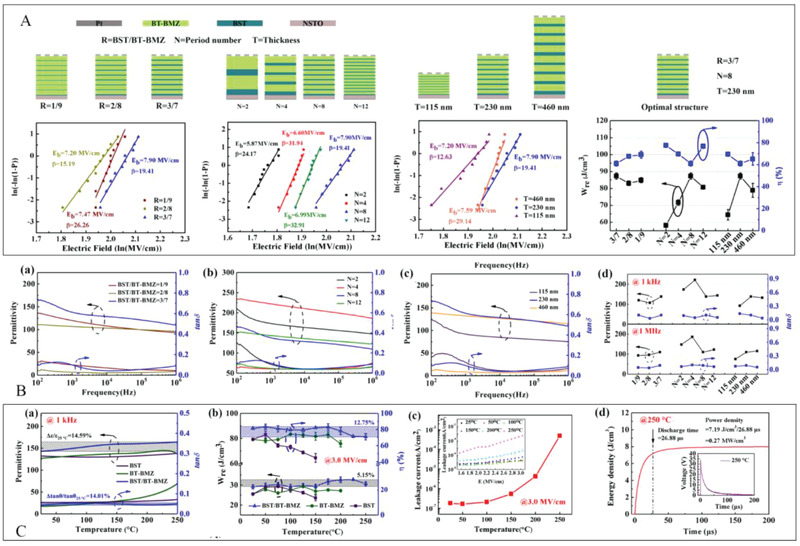
A) Schematic diagram of the multilayer structure and the corresponding breakdown strength and energy storage characteristics. B) Frequency depends on dielectric characteristics at RT and comparison of dielectric properties of different multilayer dielectrics at some certain frequency. C‐a) Dielectric properties at 1 kHz, b) energy storage density, and c) leakage current at 3 MV cm^–1^ as a function of temperature. d) Discharging characteristics at 250 °C. Reproduced with permission.^[^ ^62^ ^]^ Copyright 2020, American Chemical Society.

Ceramics with low Curie temperatures can be selected as materials for multilayer structures because they are more likely to have energy storage property stability at high temperatures. For example, combining ST with a Curie temperature of ‐103 °C^[^ ^153^ ^]^ with Bi(Mg_0.5_Zr_0.5_)O_3_ (BMZ) with a Curie temperature of ‐25 °C^[^ ^154^ ^]^ can enhance the energy storage properties of the ceramic at 250 °C to more than 30 J cm^–3^ (efficiency greater than 70%).^[^ ^62^ ^]^ As shown in **Figure** **19**A, there are three influencing parameters in total. By varying one factor by keeping two of them unchanged, the other factor is changed to investigate the effects of the thickness ratio between two types of layers, the number of repeated cycles of the laminated layers and the total thickness of the multilayer dielectric on the breakdown strength and other performance. First, three typical composition ratios, i.e., 1:9, 2:8, and 3:7, were studied to explore their breakdown behavior. It was concluded that the dielectric with the thickness ratio of 3/7 possesses the maximum breakdown strength. The effect of the number of repetitive cycles N was investigated at a fixed ratio 3:7 and an overall thickness of about 230 nm. It was observed that the breakdown strength initially increases with the increased cycle number of the stacked layers, reaching a maximum value of 7.90 MV cm^–1^ at a repetition period of 8, and decreases marginally to 6.99 MV cm^–1^ at cycle 12. Based on the above, this trend is relatively common. The following research is concerned with the effect of the total thickness of the dielectric on its breakdown characteristics. The results revealed that the breakdown strength first rises with the total thickness and hits a peak at around 230 nm, and thereafter falls gradually. This phenomenon is considered to be the consequence of the competition between the interfacial density effect and the interfacial “dead layer” effect. *U*_e_ and *η* of the multilayer film are summarized in Figure 19B. It can be observed that the multilayer film has obtained a *U*_e_ of approximately 90 J cm^–3^ and *η* of over 60% at room temperature when the thickness ratio of the two layers is 3:7, the number of cycles is 8, and the aggregate thickness is approximately 230 nm. It should be noted that bipolar hysteresis lines were also measured in this work. A comparison of its calculated results with those of the unipolar hysteresis return line reveals no major differences.

##### Doped Multilayer Ceramic Films

Doping modification is a popular and highly effective technique to improve dielectric characteristics.^[^ ^119^, ^128^, ^156^, ^157^ ^]^ Designing the dielectric into a multilayer structure by doping in the ceramic dielectric is also a valid approach to improve capacity of energy storage.^[^ ^158^, ^159^ ^]^ Through doping, the single‐layer insulation performance or polarization strength can be improved, and multilayer dielectric can be built to realize polarization and breakdown decoupling, to enhance the energy storage characteristics of dielectrics.

**Figure 20:**
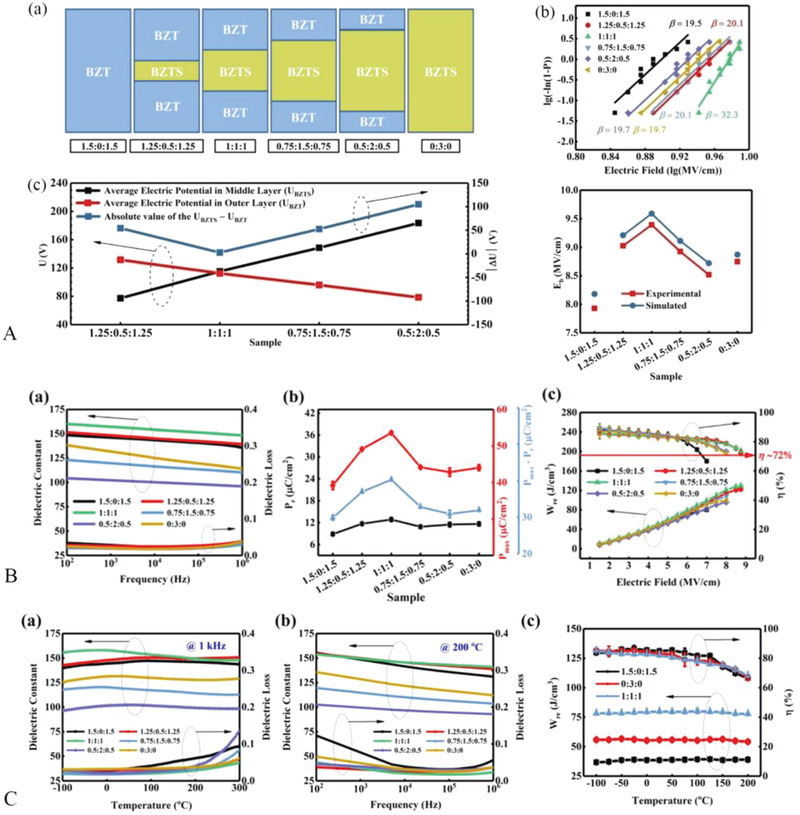
A‐a) Schematics showing the configurations of the BZT, BZTS single‐layer thin films, and sandwich‐structure thin films. b) Breakdown strength Weibull distribution and its comparison with simulation. c) The average potential of the different layers in the sandwich film, as well as the absolute value of the potential difference between the top and middle layers, calculated using a numerical integration algorithm. Electrical properties of a series of sandwich structure films at room temperature (B) and high temperature (C). Reproduced with permission.^[^ ^155^ ^]^ Copyright 2019, Elsevier.

As seen in **Figure** **20**A‐a for instance, Fan et al.^[^ ^155^ ^]^ chose BaZr_0.35_Ti_0.65_O_3_ (BZT) doped with 1 mol% SiO_2_ as the material of the middle layer, and the undoped BZT as the outer layer to prepare a composite dielectric with sandwich structure. The influence of the thickness ratio of the outside layer to the middle layer on the dielectric properties has been systematically investigated. With a fixed total thickness of the sandwich structure dielectric, *E*_b_ and shape parameter (ɛ) are extremely reliant on the thickness ratio of the inner–outer layer. Both *E*_b_ and ɛ first increase with the thickness of the inner layer and reach a maximum value with a thickness ratio of 1:1:1, and then decrease slightly. This is consistent with the simulation results. Using a numerical integration algorithm, the average electrical potential in the inner and outer layers of the interlayer film was calculated. The average electrical potential of the inner and outer layers and the difference between these two potentials (*ΔU*, *|ΔU| = |U_BZTS_*‐*U*_BZT_|) are plotted in Figure 20A‐c. As can be seen from the figure, *ΔU* is minimum at a thickness ratio of 1:1:1, which could not accelerate free electrons to a sufficiently high energy state^[^ ^160^ ^]^ and thus largely limits the growth of breakdown path and significantly improves the *E*_b_. The electrical performance of the sandwich structured dielectric at RT and high temperature are displayed in Figure 20B,C. The introduction of silica makes the conductivity of the middle layer different from that of the outer layer, and the space charge exists around interfaces, thus increasing ɛ_r_ and polarization. The multilayer dielectric with a thickness ratio of 1:1:1 has the best energy storage characteristics due to the best polarization and breakdown properties, as shown in Figure 20B‐c. In addition, its temperature stability performance is excellent (Figure 20C) (**Table** **2**).

**Table 2:** Multilayer ceramic dielectric and its related electrical properties

	Dielectric properties @RT		Thickness	*E*_b_ [MV m^–1^]	Polarization [µC cm^–2^] @RT		Energy storage density [J cm^–3^]/efficiency[%]			Refs.
Sample	*ε*_r_	Tan*δ*			*P*_m_	*P*_r_	RT	150 °C	200 °C	
Eight‐layer BT/ST	≈1500 (@18G Hz)	0.04 (@18G Hz)	480 nm	**–**	**–**	–	–	–	–	^[^ ^65^ ^]^
BT:ST 4:2	≈3800	0.01	250 µm	15.7	9	≈1	1.16	–	–	^[^ ^143^ ^]^
Eight‐layer BCT/BZT	≈215(@100Hz)	0.08(@100Hz)	100 nm	450	–	–	52.4	–	–	^[^ ^147^ ^]^
Ten‐layer BCT/BZT	280(@100Hz)	0.1(@100Hz)	200 nm	451	36	6	51.8/81.2	28/78	25.1/63.6	^[^ ^149^ ^]^
Six‐layer BZT15/BZT35	≈185(@100Hz)	0.05(@100Hz)	260 nm	830	45	10	83.9/78.4	≈70/≈73	63.2/66.9	^[^ ^98^ ^]^
Eight‐layer BST/BT‐BMZ	≈145(@1kHz)	0.01(@1kHz)	230 nm	790	35	5	≈29/≈82	≈28/≈81	≈40/≈77	^[^ ^62^ ^]^
Six‐layer NKBT/BSMT	600(@100Hz)	0.08(@100Hz)	280 nm	305.1	109	20	91/68	68/71	65/71	^[^ ^161^ ^]^
Six‐layer 0.7NBT‐0.3ST/0.6ST‐0.4NBT	650(@1kHz)	0.02(@1kHz)	190 nm	261.2	90	20	60/50	26/70	–	^[^ ^91^ ^]^
BZT/BZTS/BZT	160(@100Hz)	0.03(@100Hz)	400 nm	939	55	12	190/72	80/75	75/68	^[^ ^155^ ^]^
Four‐layer BTBZNT/BTAS	1400	0.02	70–80 µm	79	24.9	2	5/70	–	–	^[^ ^162^ ^]^
Three‐layer BNKSTT/BNT‐SNA/ BNKSTT	1500	0.05	50–60 µm	57.2	40	2				^[^ ^163^ ^]^

### 3.2 Polymer‐Based Multilayer Composite

Metallized polymer capacitors are self‐healing and noninductive, with high insulation properties, good flexibility and low density, which have widespread use in electronic communications, power electronics and pulse power systems.^[^ ^34^, ^164^, ^165^ ^]^ However, the low dielectric constant of polymers compared to ceramics in commercial dielectric capacitors results in a lower energy storage density.^[^ ^13^, ^27^ ^]^ Therefore, the first thing that needs to be addressed is how to increase the dielectric constant of polymers. The main method is to fill with high dielectric ceramic fillers. In addition, suppression of carrier transport is also of great interest. The main methods include orientation distribution of 1 and 2D fillers, wrapping the filler surface with a buffer layer, designing multilayer structures, gradient structures, etc. Among them, the construction of multilayer structures has been proved to be a simple and powerful method to optimize the energy storage performance.

#### 3.2.1 Construction of Multilayer Structures through Inorganic Filling

As mentioned above, polymer‐based dielectrics suffer from a low polarization strength/dielectric constant (<10 in most cases,) in comparison to electroceramics whose relative dielectric constant can reach to hundreds and even thousands, which is the main reason for its low energy storage density. One important solution is to design and fabricate organic–inorganic composite by adopting polymer as a matrix and high dielectric constant inorganic nanoparticles as fillers,^[^ ^166^, ^167^, ^168^ ^]^ such as lead zirconate titanate (PZT), barium titanate (BT),^[^ ^169^ ^]^ barium strontium titanate (BST) etc.^[^ ^170^ ^]^ The influence of the ceramic filler is not only to raise the dielectric constant of the composite dielectric, but may also lead to a reduction in the breakdown strength. This is not only due to the intrinsically lower breakdown strength of the ceramic filler, but also associated with the local electric field aberrations (caused by the difference in dielectric constant) between the substrate and the filler. Therefore, many works have been invested to introduce buffer layers. The buffer layer consists of materials such as titanium dioxide (TiO_2_), alumina (Al_2_O_3_), and silicon dioxide (SiO_2_) with low dielectric constant. Even so, the increase in energy storage density is still limited due to the negative coupling between breakdown strength and dielectric strength. The advantage of a multilayer structure is that it can be designed separately for different layers. So far, multilayer structures are considered to be the most effective way to achieve simultaneous improvements in breakdown and polarization. In the construction of multilayer structures using the filling method, dielectric with the same filling condition is considered as one layer.

##### Sandwich Structure Constructed by Filling

The sandwich structure is a simpler multilayer structure, i.e., the same filling situation (same type and doped filled with the same amount of nanofillers) is used in the dielectric layer close to the electrode side, and another filling case of the dielectric is used in the middle layer. The inorganic filling allows more precise control of the properties of each dielectric layer, with a large scope of operation. In a basic sandwich structure, for example, the upper and bottom layers can be filled with high dielectric constant nanofillers, so that these two layers have high dielectric constants but at the same time reduce the breakdown of the polymer. The center layer of the sandwich structure still maintains the high bulk breakdown strength of the polymer. The main consideration of this design is that the breakdown occurs after the formation of a path through the dielectric, so improving the insulation of the middle layer can effectively block the breakdown path through, thus improving the insulation of the composite dielectric. Fillers for soft layers are often chosen from particles with high dielectric constants, such as barium titanate particles,^[^ ^166^, ^171^, ^172^, ^173^, ^174^ ^]^ titanium oxide particles,^[^ ^175^ ^]^ and other high dielectric fillers^[^ ^176^ ^]^ etc. The middle “hard layer” can be doped with a small amount of nanoparticles,^[^ ^171^ ^]^ nanofibers,^[^ ^175^ ^]^ and other high insulation fillers^[^ ^177^, ^178^ ^]^ to make the insulation of the middle layer higher than that of the “soft layer.” It is important to note when the free electrons in polymer composites with small amount of nanofillers are accelerated by the applied electric field and move inside the Debye length of nanofillers, they are scattered or attracted and lose their energy, leading to a higher breakdown strength.^[^ ^179^ ^]^

**Figure 21:**
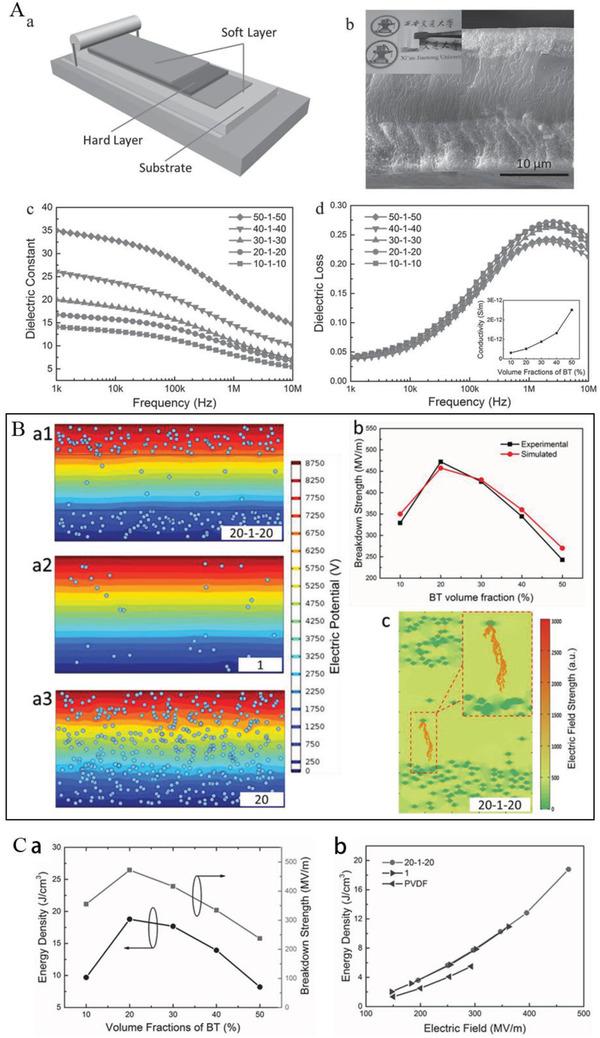
A‐a) Diagram of the manufacturing process and b) SEM image of the cross section of sandwich nanocomposites. Frequency‐dependent c) *ε*_r_ and d) tan*δ* of sandwich nanocomposites. B‐a) The distribution of electric field. b) Breakdown strength measurements versus simulation results. c) Simulation of the breakdown process. C) Energy storage density of sandwich nanocomposites. Reproduced with permission.^[^ Copyright 2015, Wiley‐VCH.

For example, as shown in **Figure** **21**A‐a,b, the BaTiO_3_/PVDF nanocomposites films with “Soft‐Hard‐Soft” sandwich structure is fabricated by solution casting method layer by layer. In the “hard layer” 1 vol% BT was chosen as nanofiller because 1 vol% BT/PVDF showed the highest breakdown strength in all nanocomposites containing a small amount of BT in BT/PVDF. A higher ceramic volume fraction (10–50 vol%) was used as two outer layers (“soft layers”) to provide high dielectric constants. The ceramic content in the soft layers was systematically adjusted to obtain a better energy storage density. A homogeneous composite dielectric with the same content was also prepared as a comparison. The dielectric properties of sandwich structure dielectrics with different filling amounts are shown in Figure 21A‐c,d, and the breakdown strength and energy storage properties are shown in Figure 21B,C. The distribution of the potential shows that in the sandwich structure, the hard layer bears a larger potential difference while the soft layer bears a smaller potential difference, which is considered to be the explanation for the increased *E*_b_. Collectively, *ε*_r_ increases with the increase of soft layer filling, while *E*_b_ grows first and then drops. And the energy storage density has the same trend as the breakdown strength. As observed in Figure 21C‐b, the energy storage density of 20‐1‐20 sandwich structure dielectric is obviously superior to the pristine PVDF and uniformly distributed dielectric.

At the same time, the initial optimization effect achieved by the sandwich structure triggers relevant. Designing the outer layer as a hard layer doped with BNNS,^[^ ^180^, ^181^, ^182^, ^183^ ^]^ or low content of nanoparticles,^[^ ^183^ ^]^ which is also a very promising design idea. The experimental results demonstrate that the designed sandwich dielectric boasts a resistivity an order of magnitude higher than that of the single‐layer composite. This is ascribed to the fact that this design avoids direct contact between the polarization layer and the electrodes, which restrains the charge injection.

**Figure 22:**
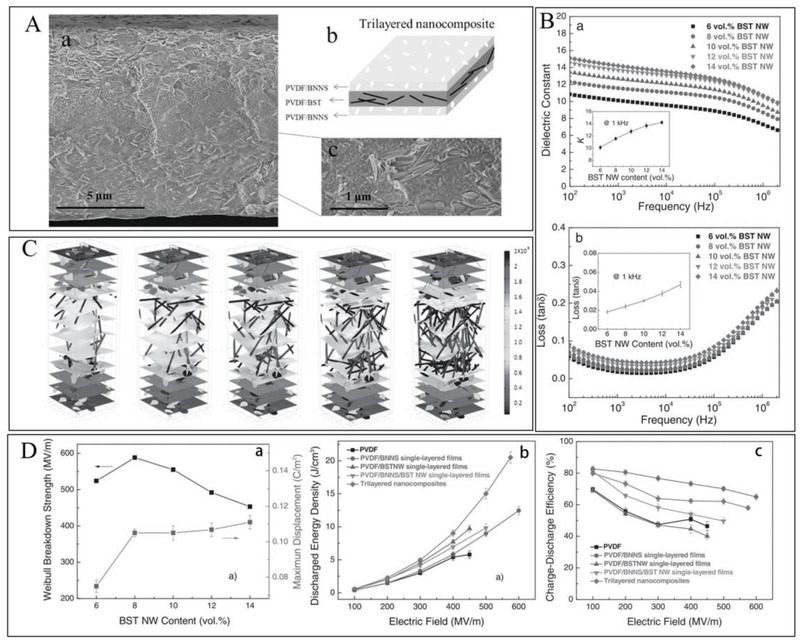
A) Cross‐sectional SEM image and schematic of “Hard‐Soft‐Hard” sandwich dielectric. B) Frequency‐dependent a) *ε*_r_ and b) tan*δ* of “Hard‐Soft‐Hard” sandwich dielectric. C) The development of electrical path in “Hard‐Soft‐Hard” sandwich dielectric with different BST NW contents under the applied *E* of 550 MV m^–1^. D‐a) Breakdown strength and maximum polarization as a function of BST content. Comparison of b) *U*_e_ and c) *η* of different structured dielectrics. Reproduced with permission.^[^ ^180^ ^]^ Copyright 2017, Wiley‐VCH.

Meanwhile, the initial optimization effect achieved by the sandwich structure triggers the related thoughts. The position of the “hard layer” has an important impact on the performance tuning. In contrast to the “soft‐hard‐soft” sequence, the dielectric was designed as a “hard‐soft‐hard” sandwich structure and its related properties were investigated. This proved to be a very promising design idea. In this case, the fillers of hard layer can be BNNS^[^ ^103^, ^104^, ^105^ ^]^ or a low content of nanoparticles.^[^ ^106^ ^]^ For example, as shown in **Figure** **22**A, the outer layer of the sandwich structure consists of PVDF doped with boron nitride nanosheets, which can provide higher breakdown strength. It is a well‐known fact that the tan*δ* under high electric field mainly comes from the tunneling current, which grows exponentially with the electric field and can significantly reduce the discharge energy density and charging/discharging efficiency. Therefore, placing charge‐blocking materials in the outer layer will effectively reduce leakage currents and conduction losses. Boron nitride nanosheets with wide forbidden band, high mechanical strength, high thermal conductivity, and low‐density characteristics are selected as fillers in the outer layer to effectively prevent leakage currents. Barium strontium titanate nanowires with high dielectric constant were selected as fillers for the intermediate soft layer to provide high *ε*_r_ for the composite. The effects of filler content on polarization, *E*_b_ and energy storage properties were systematically explored. Simulations were performed to model the formation of breakdown paths to verify the experimental breakdown results (Figure 22C). From the simulated results, the following conclusions can be drawn that the redistribution of *E* in the three‐layer nanocomposites suppresses the development of breakdown path. The dielectric characteristics of the three‐layer structured nanocomposites with different BST nanofibers contents in the central layer are shown in Figure 22B. Both *ε*_r_ and tan*δ* increase steadily with the increase of BST NW content. From the comparison graph of energy storage density and efficiency (Figure 22D‐b,c), it can be noted that *U*_e_ of the sandwich structured dielectric is the highest while the energy storage efficiency is second only to the single‐layer BNNS‐doped dielectric. The increase in *U*_e_ is attributed to the increase in breakdown strength and polarization. The increase in *η* is attributed to the decrease in residual polarization, which is caused by the fact that PVDF consists mainly of non‐polar phases (*α*, *γ*).

**Figure 23:**
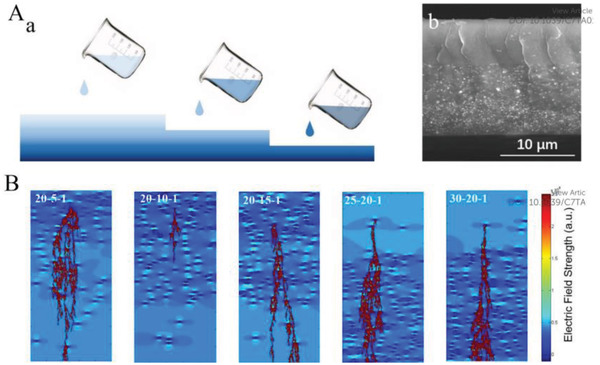
A) Manufacturing process diagram a) and cross‐sectional SEM image b) of gradient layered nanocomposites. B) The breakdown path development and field distribution in gradient layered nanocomposites with different filling amounts. Reproduced with permission.^[^ ^186^ ^]^ Copyright 2017, The Royal Society of Chemistry.

In addition, other topologies have been systematically investigated.^[^ ^184^, ^185^ ^]^ For example, as shown in **Figure** **23**, the filling content of the filler in the three‐layer structure is designed as a gradient, and the results show that the breakdown strength is also increased for the following reason: with high frequency AC fields, the local *E* magnitude is inversely correlated with the magnitude of the dielectric constant at that position, causing a lower electric field at locations with higher fill concentrations. The breakdown path from the positive pole downward will experience a change in electric field from high to low, so that the excitation energy of the electric field on the associated particles also becomes lower and the breakdown path develops slower, thus increasing the breakdown strength.

**Figure 24:**
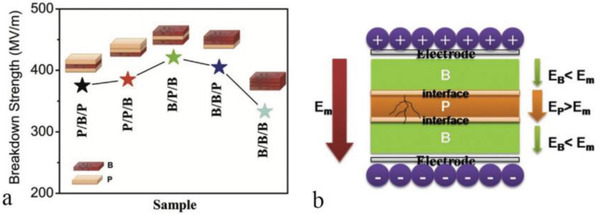
A) Comparison of breakdown characteristics of sandwich dielectrics with different topologies. B) Schematic diagram of the enhanced breakdown strength in B/P/B. Reproduced with permission.^[^ ^184^ ^]^ Copyright 2017, Elsevier.

In **Figure** **24**, the energy storage characteristics of dielectrics with different combinations of soft and hard layers are systematically compared. The results show that the “soft‐hard‐soft” combination has the best breakdown performance in this system.

**Figure 25:**
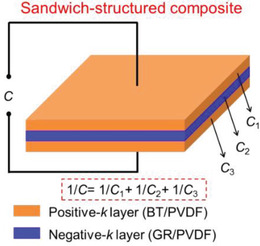
Schematic of sandwich‐structured composites with a negative‐k layer. Reproduced with permission.^[^ ^189^ ^]^ Copyright 2017, The Royal Society of Chemistry.

Materials with negative dielectric constants are very interesting, and not much research has been done in this area, but some progress has been made in recent years.^[^ ^187^, ^188^ ^]^ For example, as shown in **Figure** **25**, polymer composites with sandwich structure made up of alternating positive and negative dielectric constant layers were fabricated.^[^ ^189^ ^]^ The overall *ε*_r_ of the sandwich structure was enhanced using the negative dielectric constant layer. A multilayer structured dielectric can be theoretically equivalent to a capacitor composed of multiple series capacitors. According to the equation for series capacitance, in general, capacitors in series are positive, so the equivalent capacitance will be smaller than any one of them. Interestingly, if one or several of them become negative while the remaining capacitance remains positive, an enhanced equivalent will be obtained. Based on this idea, the polymer‐based composite dielectric consisting of graphite particles dispersed in a polymer matrix is made into a negative dielectric layer with a negative weak dielectric constant that matches well with the dielectric constant of the positive layer. In contrast to conventional multilayer composites, these three‐layer composites are able to counterbalance conflicting parameters to produce remarkably high dielectric constants without sacrificing low tan*δ* and high *E*_b_. It has been empirically proven that this proposed new composite has a greater energy storage capacity than the conventional multilayer composites. In summary, the introduction of negative dielectric constant materials into the structure to construct high dielectric constant composites is a relatively innovative and effective design concept (**Table** **3**).

**Table 3:** Relevant electrical properties of sandwich structured polymer‐based composite dielectrics

Sample			Dielectric properties @RT		*E*_b_ [MV m^–1^]	Polarization [µC cm^–2^] @RT		Energy storage density [J cm ^–3^]/Efficiency [%]			Refs.
Frist	Second	Third	*ε*_r_	Tan*δ*		*P*_m_	*P*_r_	RT	150°C	200°C	
BT‐NPs/PVDF	BT‐NFs/PVDF	BT‐NPs/PVDF	10(@100Hz)	0.016(@100Hz)	453	–	–	9.72/‐	–	–	^[^ ^166^ ^]^
BT/PVDF	PVDF	BT/PVDF	17(@1kHz)	0.04(@1kHz)	472.1	–	–	18.8/65	–	–	^[^ ^171^ ^]^
TO NPs/PVDF	BSBT NFs/PVDF	TO NPs/PVDF	13.7(@1kHz)	0.03(@1kHz)	300	8.75	2	8	–	–	^[^ ^175^ ^]^
BT/PVDF	PVDF	BT/PVDF	12(@1kHz)	0.04(@1kHz)	410	–	–	17/63	–	–	^[^ ^190^ ^]^
BNNS/PVDF	BST/PVDF	BNNS/PVDF	12.3(@100Hz)	0.07(@100Hz)	580	10.7	–	20.5/57	–	–	^[^ ^180^ ^]^
BNNS/c‐BCB	BT/c‐BCB	BNNS/c‐BCB	5.4(@100Hz)	0.02(@100Hz)	–	–	–	–	4/80	0.8/	^[^ ^181^ ^]^
NaNbO_3_/PVDF	PVDF	NaNbO_3_/PVDF	13(@1kHz)	0.025(@1kHz)	402.6	–	–	13.5/65	–	–	^[^ ^182^ ^]^
1 vol% BT/P(VDF‐HFP)	9 vol% BT/P(VDF‐HFP)	1 vol% BT/P(VDF‐HFP)	11(@1kHz)	0.045(@1kHz)	526	13.5	0.5	26.4/72	–	–	^[^ ^183^ ^]^
BT/PVDF	PVDF	BT/PVDF	10.1(@1kHz)	0.02(@1kHz)	390	12	7.5	7.02/‐			^[^ ^184^ ^]^
P(VDF‐HFP)	4 vol% BTNFs/P(VDF‐HFP)	15 vol% BT/P(VDF‐HFP)	10.1(@1kHz)	0.03(1kHz)	510	9.5	1.5	17.6/71.2	–	–	^[^ ^185^ ^]^
BT/PVDF	GR/PVDF	BT/PVDF	92.5(@1kHz)	0.029(@1kHz)	188.9	–	–	16/‐	–	–	^[^ ^189^ ^]^
BT/PVDF	PVP	BT/PVDF	–	–	447.4	–	–	6.2/‐	–	–	^[^ ^191^ ^]^

##### Laminated Multilayer Structure Constructed by Filling

As mentioned earlier, in polymer‐based dielectrics, the interface between layers (vertical to the applied electric field) has beneficial effects on the performance of the material, then, if the number of such interfaces increases, multilayer dielectrics should exhibit a superior performance.^[^ ^192^ ^]^ Taking advantage of the fact that inorganic filler filling is very easy to modulate, more complex multilayer structures are designed to come out with improved energy storage characteristics. By regulating the content and type of inorganic filler filling, the dielectric layers with different filling conditions will be designed at different locations to make full use of the performance characteristics of different layers to provide the breakdown strength and polarization of the dielectric synergistically.

**Figure 26:**
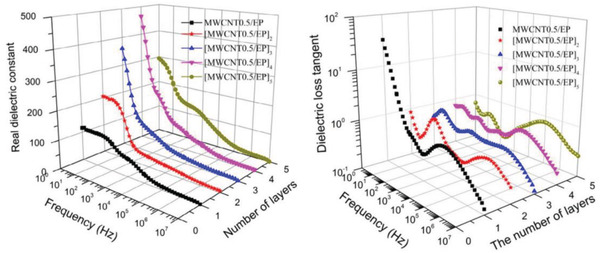
Variation of dielectric properties of multilayer dielectrics with frequency and number of layers. Reproduced with permission.^[^ ^193^ ^]^ Copyright 2015, Elsevier.

In multilayer structures (with excess of 3 layers), exploring the law of the influence of the number of layers on the dielectric feature is often a key direction of interest for researchers. Chi et al. prepared the multilayer high dielectric constant carbon nanotube/epoxy composites and explored the dielectric properties.^[^ ^193^ ^]^ **Figure** **26** illustrates the dependence of the dielectric constant on frequency for a multilayer composite ([MWCNT/EP]*x*, where *x* refers to the number of layers) based on MWCNT‐doped EP. As can be observed, *ε*_r_ initially grows with increased number of layers and decreases when the number of composite layers exceeds 4. Strikingly, the highest dielectric constant obtained from a 4‐layer dielectric is about four times higher than that of an ordinary single‐layer composite. Figure 26 also shows the superposition of the dependence of tan*δ* on frequency for composites with different number of layers. Contrary to single‐layer dielectrics, multilayer dielectrics exhibited remarkably low dielectric dissipation in the measured frequency range. The single‐layer dielectric shows a significantly high dielectric loss tangent due to conductivity loss in the very low‐frequency range, which does not exist for the other materials. In conclusion, by constructing multilayer structures and optimizing the structural parameters, the dielectric performances can be controlled in a wide range.

**Figure 27:**
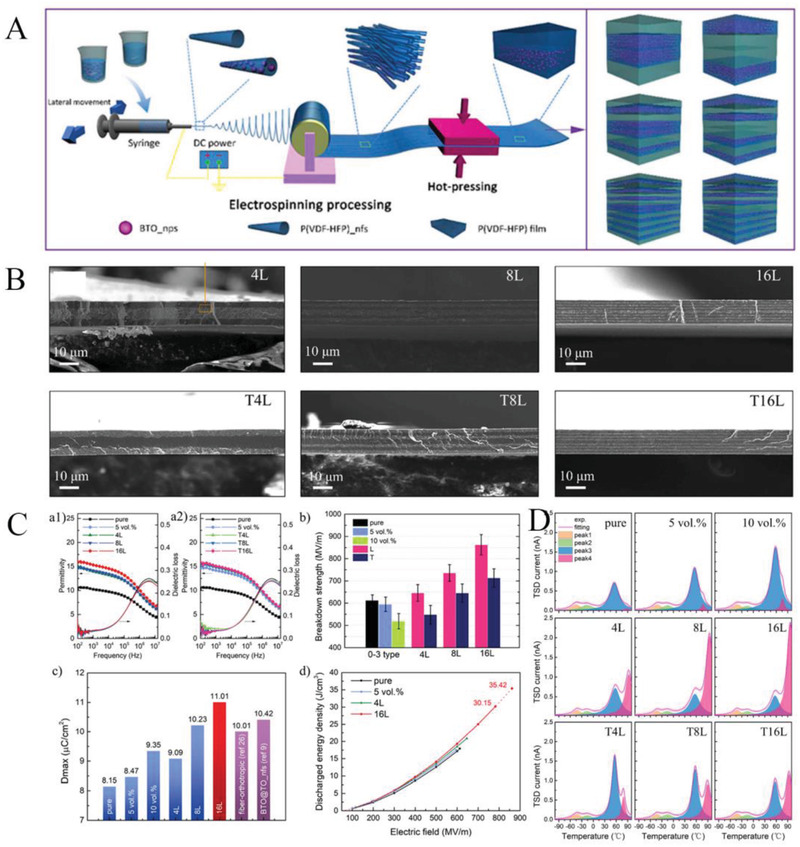
A) Schematic diagram of the preparation of multilayer polymer nanocomposites using electrostatic spinning (left) and six structures of multilayer dielectric (right). B) SEM images, C) electrical performance, and D) TSDC spectra for multilayered dielectric. Reproduced with permission.^[^ ^192^ ^]^ Copyright 2019, Elsevier.

The effect of multilayer structures on energy storage performance has been extensively studied. Shen et al. prepared multilayered polymer nanocomposites has a high integrity interface which is prepared through a step‐by‐step process of electrospinning‐hot pressing‐hot quenching. The dielectric layers are alternately distributed, i.e., one layer doped and one layer undoped (see **Figure** **27**A). P(VDF‐HFP) is chosen for the matrix and BaTiO_3_ nanoparticles (≈50 nm) for the filler. Two multilayer composite dielectric materials with opposite filler concentration distribution structures (in one structure, the layer doped with high dielectric constant ceramic particles is close to the electrode, while in the other structure, the pristine polymer layer is close to the electrode) of the same total thickness were prepared as shown in Figure 27A,B. The electrical properties of the multilayer nanocomposites are summarized in Figure 27C. It appears that the number of layers in the multilayer structure has little effect on the dielectric properties of the dielectric, since the multilayer composites exhibit a very slow and small increase in the dielectric constant and a decrease in the dielectric loss as the number of layers increases. However, as shown in Figure 24B‐b, the increase in the number of layers has a significant enhancement on the breakdown strength of the multilayer dielectric. Due to this enhancement on breakdown, the maximum polarization intensity is also enhanced. In addition, increasing the interfacial polarization strength may also contribute. As a logical consequence of the enhancement of Pm and Eb, the multilayer composite dielectric exhibits enhanced energy storage properties. Figure 27D shows the TSDC spectra of multilayer nanocomposites. The peak at ≈58 °C (blue) is believed to be due to depolarization of the charge injected from the electrode while the discharge peak at 75–95 °C (pink) is attributed to depolarization of the ion. The area and central temperature of the blue and pink peaks are summarized as shown in Figure 27C‐b. For the monolayer nanocomposites, the region of the blue peak shows an area increase with the filler content, demonstrating increased charge injection when filled with nanofillers with high *ε*_r_. While in multilayer structures, the area of blue peak decreases with increasing number of layers, demonstrating that the charge injection from the electrodes is suppressed. The area of the pink peak increases with the increase of the number of layers, explaining the better energy storage performance of the multilayer structure from the side.

In addition to filling by particles, constructing multilayer nanostructures by filling 1D nanofibers is also a good choice. The fiber‐doped five‐layer structures were prepared and their related electrical properties were investigated by Zhang et al.^[^ ^194^ ^]^ The structure has been found to increase the breakdown strength while maintaining low losses.

##### Constructing Gradient and Orthogonal Multilayer Structures

Owe to the large differences in *ε*_r_ and conductivity(*σ*) between different layers in the stack, large electric field distortions often exist in the dielectric. Establishing a gradient structure with gradually changing filler can achieve smooth transition and thus electric field homogenization.^[^ ^195^ ^]^

**Figure 28:**
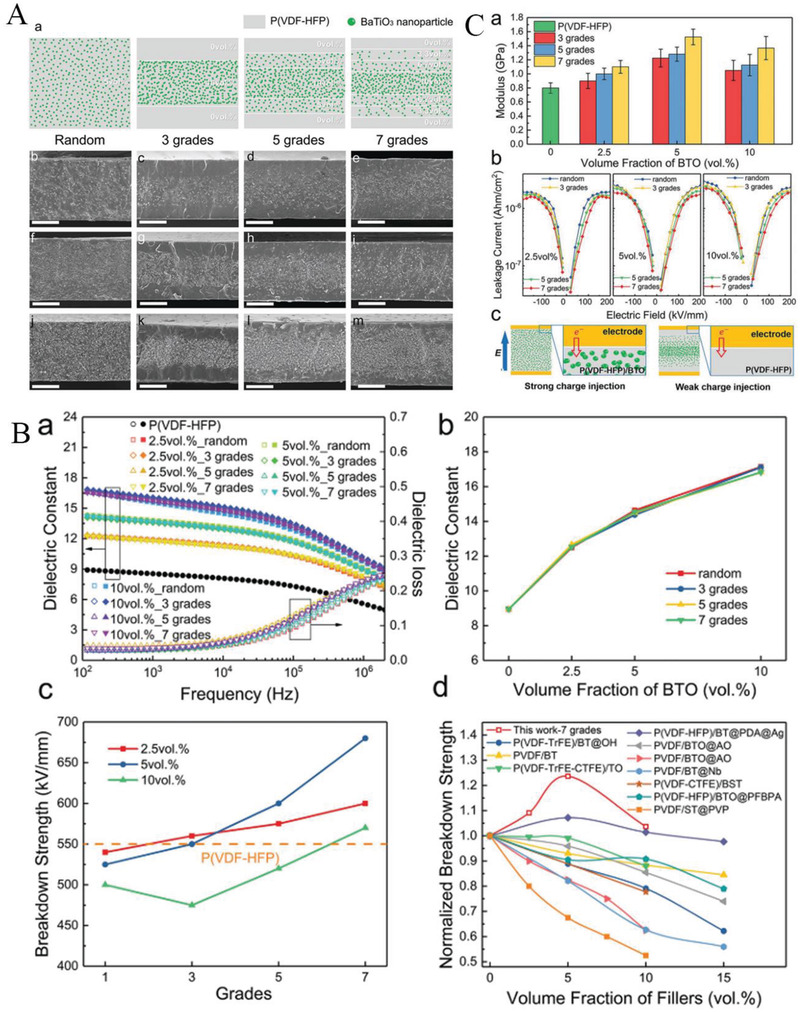
A) Diagram and SEM images of gradient nanocomposites. B) Dielectric and breakdown properties of gradient composite dielectric. C‐a) Out‐of‐plane Young's modulus, b) variation of leakage current density with electric field, and c) schematic illustration of metal/dielectric interfaces for nanocomposites with random and gradient distribution of nanoparticles(c). Reproduced with permission.^[^ ^196^ ^]^ Copyright 2019, Wiley‐VCH.

Thin layers of P(VDF‐HFP)‐based composite dielectrics with different volume fractions of BaTiO_3_ nanoparticles were pressed into multilayer structures in a certain order by Zhang et al.^[^ ^196^ ^]^ Among them, the multilayer structures were designed as random, 3‐grades, 5‐grades, and 7‐grades to examine the impact of smoothness on performance. What can be observed is that the dielectric constant increases with increasing filling content, while there is only a small difference in *ε*_r_ between the graded multilayer structure and the randomly distributed structure. However, the multilayer structure (number of layers) has a significant effect on the dielectric breakdown strength. By adjusting the BT nanoparticles from random to gradient distribution, the breakdown strength starts to increase. The variation of breakdown strength (normalized to the original polymer) with nanofiller loading for various polymer composite dielectrics doped with spherical nanofillers is collected and presented in **Figure** **28**B‐d. It can be seen that the breakdown strength of the composite dielectric can be enhanced in the multilayer structure, even with the doping of ceramics. There are two main reasons for it, one is the increase in Young's modulus. The mechanical properties along the out‐of‐plane direction were investigated and recorded in Figure 28C‐a. It is known that electromechanical breakdown occurs when the electrostatic stress induced by the electric field reaches a critical value to overcome the mechanical stress, and a higher modulus of elasticity usually indicates a stronger electrostatic force tolerance.^[^ ^197^ ^]^ It can be seen that the Young's modulus along the out‐of‐plane direction increases continuously as the number of grades of gradient‐structured nanocomposites increases. The other reason is the decrease in leakage current. The leakage current density associated with thermal breakdown is characterized and plotted in Figure 28Cb. It can be seen that the multilayer structure contributes to the reduction of the leakage current, which is mainly due to the lower local charge at the contact electrodes compared to the uniform filling and the weaker charge injection in the gradient structure.

**Figure 29:**
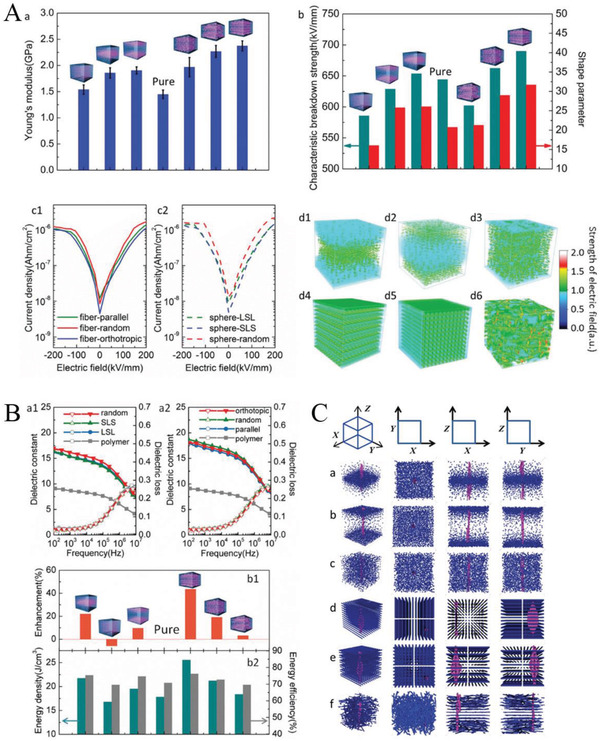
A) Mechanical and electrical properties of different nanocomposites and simulated electric field distribution by phase‐field method. B) Dielectric and energy storage properties. C) The final steady state of the breakdown path evolution by phase‐field simulations. Reproduced with permission.^[^ ^198^ ^]^ Copyright 2018, Wiley‐VCH.

Based on the above research, Shen et al. designed a gradient multilayer structure with oriented fiber fillers with better breakdown performance, and also designed a structure in which fiber fillers are arranged orthogonally.^[^ ^198^ ^]^ Composite dielectrics with oriented, orthogonal and randomly distributed nanofibers were prepared. Young's modulus, leakage current and finite element simulations for a series of films are shown in **Figure** **29**A. Compared with fiber‐random nanocomposite, fiber–orthotropic and fiber–parallel nanocomposites exhibit suppressed Young's modulus and leakage current density. Secondly, for the nanocomposites with random distribution of spheres or fibers, substantial inhomogeneous distribution of *E* is observed, which leads to the easy formation of networks of breakdown paths hence increased leakage current. While for the nanocomposites filled with aligned nanofibers, the well‐aligned nanofibers help to mitigate the concentration of local electric field. The dielectric, breakdown and energy storage properties are shown in Figure 29B. It can be observed that there is not much difference in the dielectric properties of different structures, while there is a large difference in the energy storage properties, and the trend is basically consistent with the breakdown variation. The composite dielectric with orthogonal distribution of fibers has the highest *U*_e_ and *E*_b_. This phenomenon can be explained by phase field simulations. In Figure 29C, it can be seen that the breakdown path is the most tortuous in the composite dielectric with orthogonal distribution of fibers, which leads to an increase in breakdown time and an increase in breakdown strength.

**Figure 30:**
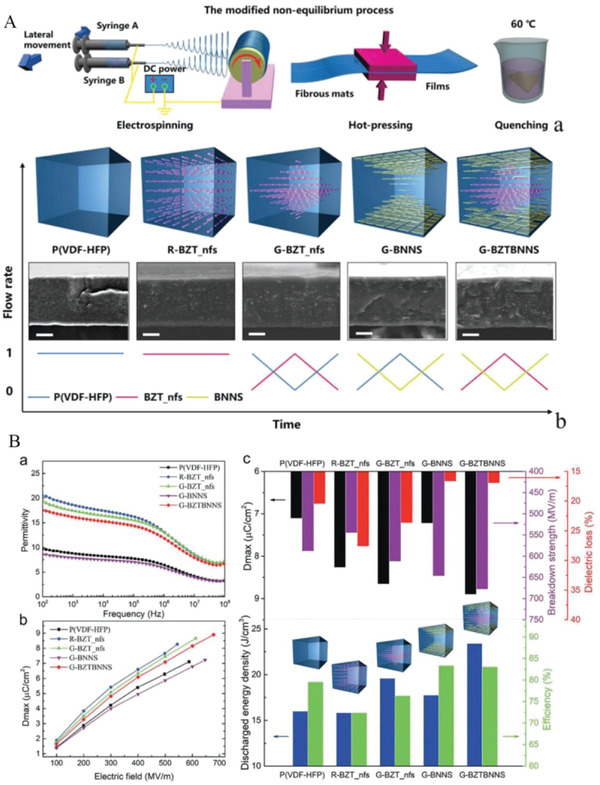
A) Preparation and structure schematic. B) Key electrical properties of gradient composite dielectric. Reproduced with permission.^[^ ^199^ ^]^ Copyright 2018, Wiley‐VCH.

Further, the introduction of a 2D material capable of enhancing breakdown into the gradient structure has also been explored, which increases the upper limit of the efficiency of the doped multilayer composite dielectric. Nanocomposites with advanced interpenetrating gradient architectures were engineered by co‐filling PVDF matrix with barium zirconium titanate nanofibers (BT NFs) and hexagonal boron nitride nanosheets (BNNS), as depicted in **Figure** **30**A. In order to study the influence of BNNS content on dielectric loss, a G‐BNNS film with only BNNS nanofillers and a reverse gradient structure (contrary to the structure of G‐BZT_nfs) was also prepared. The dielectric properties, high field polarization characteristics, and breakdown characteristics of the composite dielectric are shown in Figure 30B. The existence of BZTNFs improves the polarization of the dielectric. The existence of BNNS not only reduces the dielectric loss, but also increases the *E*_b_ and *η*, but slightly reduces the dielectric constant. The coexistence of the two composite dielectrics shows excellent polarization, *E*_b_ and *η* synergistically improved characteristics. In a short, the proposal structure is very effective in improving energy storage characteristics.

#### 3.2.2 Heterogeneous Multilayer Structure

As research continues, improving the reliability of the current research dielectric (i.e., energy storage efficiency) is found to be a sure way to improve commercial products. Because the pursuit of *U*_e_ and ignore the consequences of *η* is not only a waste of energy, but also cause thermal accumulation and thus accelerate thermal breakdown failure, greatly reducing the service life of the dielectric.^[^ ^200^ ^]^

For a long time, PVDF and its binary and terpolymers have been used as substrates for designing high performance energy‐storage dielectric because of PVDF's high *ε*_r_ and excellent processability.^[^ ^201^, ^202^, ^203^, ^204^ ^]^ However, PVDF and its binary and terpolymers have higher dielectric loss and energy storage efficiency compared to commercial dielectric BOPP. PVDF exhibits the bulk conductivity (i.e., ≈10^–9^ S m^–1^) that is at least eight orders of magnitude higher than that of benchmark BOPP (i.e., ≈10^–17^ S m^–1^).^[^ ^205^, ^206^ ^]^ Although researchers have modified PVDF with doping, such as doping with a small amount of oriented nanofibers, BNNS with high band gap width, alumina and titanium oxide with high breakdown strength, there is still a big gap with the high efficiency of BOPP of about 90%.

Therefore, improvements to the substrate are necessary and important. For example, it is relatively simple to blend different materials, such as polyamide (PA),^[^ ^207^ ^]^ poly(methyl methacrylate) (PMMA), polyimide (PI),^[^ ^208^ ^]^ polystyrene (PS), polypropylene (PP), and PVDF, to obtain a composite with better properties. Or heterogeneous multilayer structural dielectric can be prepared by techniques such as layer‐by‐layer scraping, co‐extrusion and electrostatic spinning. It is not difficult to imagine that the interface between layers in a heterogeneous multilayer dielectric is vertical to the direction of the electric field, while in a co‐blended dielectric it is not. Therefore, heterogeneous multilayer dielectrics show unique properties as a substrate.

##### All‐Organic Heterogeneous Multilayer Structure

The use of different polymers to construct multilayer structural substrates and the introduction of multiple polymer–polymer heterogeneous interfaces in the dielectric can give rise to many interesting variations. For example, more simply, ferroelectric polymers and linear polymers were prepared as three‐layer dielectrics. Wang et al. prepared a series of PMMA/P(VDF‐HFP) monolayer, bilayer, and trilayer dielectrics.^[^ ^209^ ^]^ By utilizing the complementary properties of PMMA and P(VDF‐HFP), high energy density and excellent charge‐discharge efficiency can be achieved simultaneously. The outcome revealed that *U*_e_ and *η* rises with increasing layer number, which demonstrates the advantages of a multilayer structure. The introduction of PMMA reduces the dielectric loss and increases the DC resistivity by an order of magnitude. This means that the thickness of PMMA layers can be adjusted to achieve greatly reduced dielectric losses and greatly improved electrical insulation properties. Moreover, the introduction of PMMA narrows the D–E loops significantly, which indicates that the high field energy loss decreases as the PMMA layer is increased. The increase in resistivity and the corresponding decrease in energy loss leads to a significant simultaneous increase in discharge energy density and charge‐discharge efficiency of the three‐layer dielectric. The excellence of this structure has also been recently demonstrated in a study by Jang et al.^[^ ^210^ ^]^

Eric Baer et al. have been working on co‐extruded multilayer energy storage dielectrics.^[^ ^51^, ^79^, ^80^, ^81^, ^82^, ^211^, ^212^, ^213^, ^214^, ^215^, ^216^, ^217^ ^]^ The number of dielectric layers has been achieved by melt coextrusion to 256 layers. Laminated films were prepared by combining polymer P (VDF‐HFP) with a high dielectric constant and polymer polycarbonate (PC) with a high breakdown strength, and the results indicated that this structure led to a significant enhancement of breakdown strength.^[^ ^216^ ^]^ This was followed by a range of studies on its dielectric and energy storage characteristics.^[^ ^80^ ^]^

**Figure 31:**
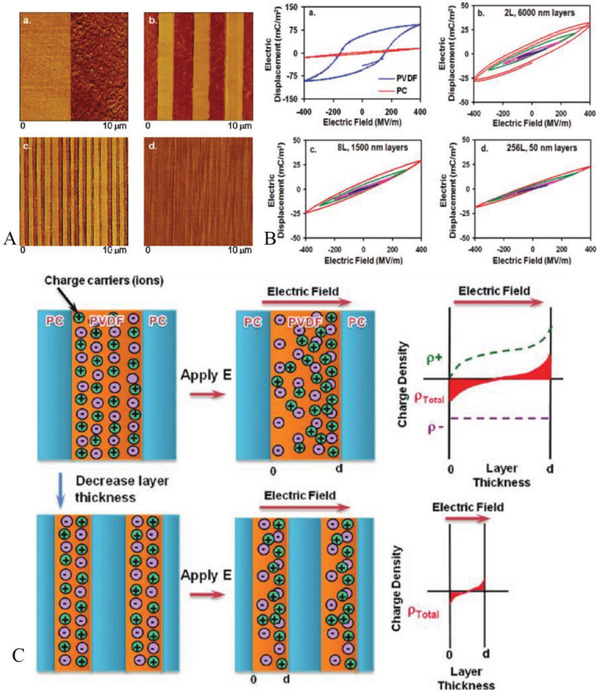
A) AFM phase images and B) *D‐E* loops of PC/PVDF layered dielectric. C) The limiting effect of layer thickness on ion migration. Reproduced with permission.^[^ ^82^ ^]^ Copyright 2012, American Chemical Society.

For instance, as shown in **Figure** **31**, the effect of multilayer structures on low‐ and high‐field losses was investigated. As the number of layers rises (the thickness of the monolayer dielectric decreases), the dielectric loss drops and the *D‐E* loops become narrow and long, which is benefit to the improvement of energy storage properties. Next, in analyzing the causes of this phenomenon, it was found that no dipole flipping occurs inside the PVDF layer and the reason could be the low local electric field inside it. It can be inferred from the *D‐E* loops of the multilayer dielectric that ion migration rather than dipole switching is to blame for the dissipation in PVDF (because the *D‐E* loops are narrow compared to pristine PVDF). The reason for the presence of these ions is analyzed as follows: PVDF is synthesized by suspension polymerization, therefore the ionic impurities should come from surfactant residues. The experimental data showed that by reducing the thickness of the PVDF layer to a few tens of nanometers, the low and high field losses were substantially reduced. In work 174, the thickness at which ion transport losses in PVDF can be reduced is investigated and an attempt is made to predict this thickness using simulation.^[^ ^217^ ^]^

**Figure 32:**
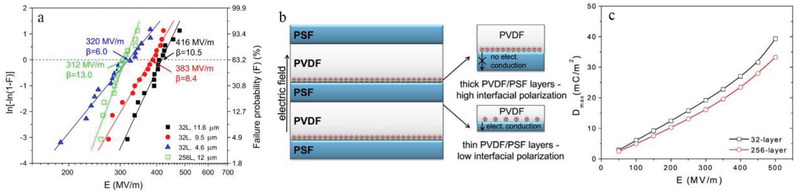
a) Weibull plots of breakdown strength. b) Schematic of the Maxwell‐Wagner‐Sillars interfacial polarization in PVDF layers and subsequent electronic conduction in PSF layers. c) *D*_max_ as a function of *E*. Reproduced with permission.^[^ ^218^ ^]^ Copyright 2014, Elsevier.

Next, the effect of layer thickness on the insulation properties of PSF/PVDF multilayer dielectric with fixed composition was investigated. As shown in **Figure** **32**a, it was found that reducing the thickness of the monolayer also reduced *E*_b_ of the multilayer dielectric which can be explained by the Maxwell‐Wagner‐Sillars interfacial polarization in the PVDF layers and subsequent electronic conduction in the PSF layers. When the more conductive PVDF layer is thick, more space charges are available to be polarized. When the insulating PSF layer is thick, internal electronic conduction is not allowed. As a result, the Maxwell‐Sillar‐Wagner interfacial polarization will be high and the accumulated charges at interfaces serve as effective traps to prevent electronic conduction. On the contrary, When PVDF and PSF layers are very thin (less than 100 nm), the interfacial polarization will be low due to fewer available space charges in PVDF layers and electronic conduction through thin PSF layers. Consequently, the overall conductivity will be relatively high, resulting in reduced breakdown strength and shorter lifetime.

**Figure 33:**
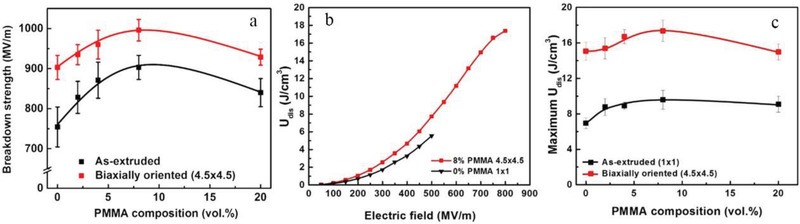
a) Dielectric breakdown strength versus PMMA composition. b) The relationship between discharge energy density and polarization electric field. c) The relationship between the maximum discharge energy density and PMMA composition. Reproduced with permission.^[^ ^219^ ^]^ Copyright 2016, American Chemical Society.

Except for fusion coextrusion of two‐layer dielectric, a three‐component multilayer films with PMMA as an intermediate layer is also designed.^[^ ^219^ ^]^ PMMA, a polymer compatible with both P(VDF‐HFP) and PET, was selected to improve the interfacial adhesion between PET and P(VDF‐HFP) layers. The experimental results demonstrate that in the prepared multilayer dielectric, there are more oriented fibrous crystals in the P(VDF‐HFP) layer and the amorphous phase is confined. There are more rigid amorphous phases in the PET layer. These changes reduce the loss and increase the breakdown strength (**Figure** **33**).

**Figure 34:**
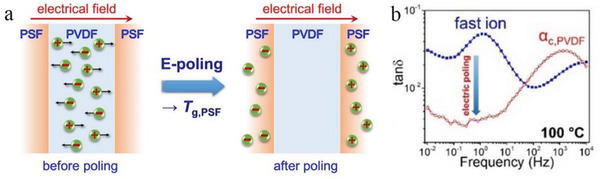
a) Movement of ions during polarization. b) Frequency dependence of dielectric loss before and after electric poling. Reproduced with permission.^[^ ^220^ ^]^ Copyright 2018, American Chemical Society.

According to the results of the above two studies, there is a contradiction between the need for a thinner PVDF layer to reduce the ionic conduction loss in PVDF and the need for a thicker layer to obtain a higher breakdown strength. Moreover, it is known that increasing the linear polymer content decreases the dielectric polarization and thus affects the energy storage density. A feasible strategy is suggested in work^[^ ^220^ ^]^ to address this challenge. Impurity ions can migrate from the PVDF layer to the PSF layer by imposing a DC electric field at a slightly lower temperature than the PSF glass transition temperature (165 °C). After cooling, the impurity ions are effectively frozen in the glassy PSF layer so that the ion conduction losses can be diminished successfully (**Figure** **34**).

**Figure 35:**
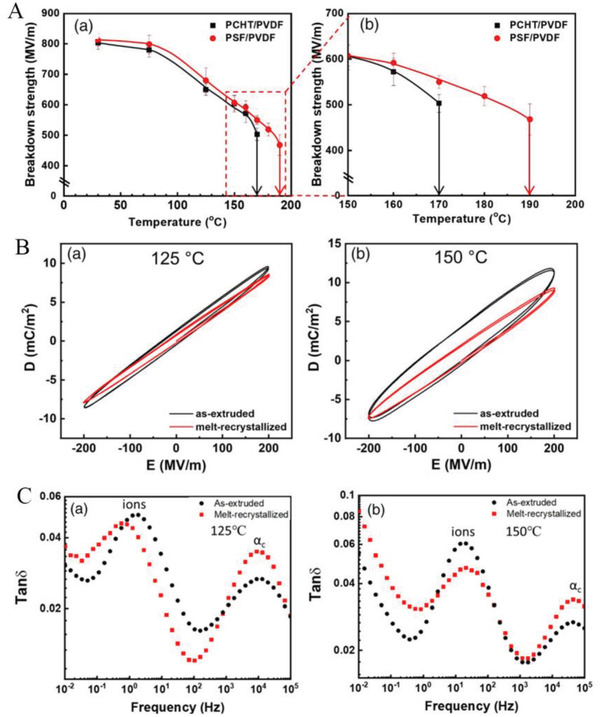
A‐a,b) Variation of *E*_b_ of multilayer dielectrics with temperature. B‐a,b) *D‐E* loops and C‐a,b) dissipation coefficients for PSF/PVDF multilayers extruded and melt recrystallized at high temperatures. Reproduced with permission.^[^ ^79^ ^]^ Copyright 2019, John Wiley and Sons.

Multilayer energy storage dielectrics for high temperature applications were further explored and designed.^[^ ^79^ ^]^ High‐*T*_g_ polycarbonate (HTPC, *T*_g_ = 165 °C) and polysulfone (PSF, *T*_g_ = 185 °C) multilayer dielectrics laminated with PVDF were prepared, respectively, and the breakdown strength versus temperature shows that at temperatures below 170°C, the HTPC/PVDF multilayer dielectrics show comparable to the PSF/PVDF multilayer dielectrics in terms of breakdown strength and hysteresis loss. At temperatures above 170 °C, PSF/PVDF has better high temperature insulation properties due to the high *T*_g_ of PSF. After recrystallization edge‐oriented crystals can be achieved due to the nano‐constraining effect. Oriented crystals provide a “blocking effect” on ions, limiting the mobility of ions. Therefore, the restricted crystallization exhibits reduced hysteresis by obtaining oriented PVDF crystals (**Figure** **35**).

The superiority of multilayer structures compared to blends was also systematically explored.^[^ ^221^, ^222^ ^]^ Due to the limited space, it will not be repeated here.

##### Heterogeneous Multilayer Structure with Inorganic Fillers

**Figure 36:**
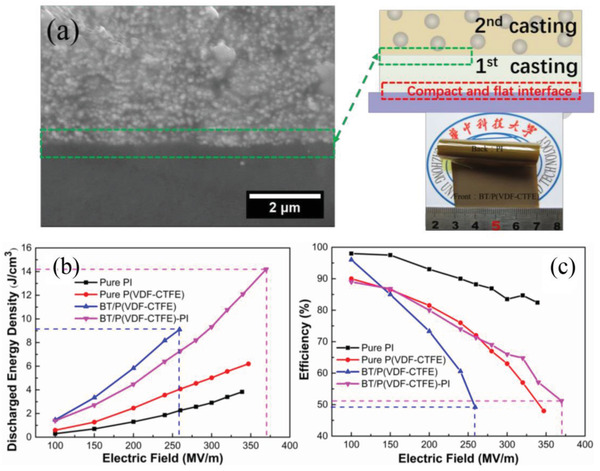
a) The cross‐section SEM image of the bilayer heterostructure BT/P(VDF‐CTFE)‐PI nanocomposite, photo of the bilayer nanocomposite film is given in inset. b,c) Variations of energy storage density and efficiency for BT/P(VDF‐CTFE)‐PI nanocomposite. Reproduced with permission.^[^ ^223^ ^]^ Copyright 2018, Elsevier.

In all‐organic multilayer structured dielectrics, the performance can be adjusted in a smaller range because the components are fixed. Therefore, many researchers have doped inorganic nanoceramic fillers to construct heterogeneous multilayer nanocomposite dielectrics.^[^ ^88^ ^]^ Generally, high dielectric constant ceramic fillers are incorporated into ferroelectric polymers.^[^ ^224^ ^]^ Zhang et al. overcome the corrosion at interface during preparation procedure of PVDF‐based multi‐layered composite dielectrics, prepared linear polymer PI and ferroelectric P(VDF‐TrFE) bilayer heterostructure.^[^ ^223^ ^]^ This structure improves the *E*_b_ and tan*δ* due to the presence of PI layer. In addition, the interfacial polarization due to the difference in conductivity between the two layers increases the polarization strength of bilayer dielectric. As shown in **Figure** **36**, this structure obtains a high energy storage density.

In addition, drawing on the experience of the results already achieved with sandwich structures in the previous section, the preparation of different polymers into sandwich structures has also proven to be a facile and efficacious methodology for improving the energy storage capacity of dielectrics. This method can effectively reduce the bending phenomenon in double‐layer dielectric due to the difference in mechanical properties of the polymer.

Further, multilayer heterogeneously doped energy storage dielectrics have also been shown to have better energy storage properties.^[^ ^225^, ^226^, ^227^ ^]^ Compared to doped multilayer dielectrics, heterogeneously doped multilayer dielectrics have an additional polymer–polymer interface, which is capable of blocking the breakdown path and breakdown path, as described earlier, and brings together the advantages of at least two polymers. In an all‐organic multilayer dielectric, since the dielectric constant and conductivity of each dielectric are fixed, the ratio of the *E*_b_ of the different layers is also fixed. When the dielectrics are not properly matched, there will be a situation where one dielectric reaches its breakdown strength first, while the other does not reach its breakdown strength. Through doping regulation, a reasonable distribution of electric field can be implemented to improve the *E*_b_ and features of the dielectric.

### 3.3 Inorganic–Organic Multilayer Dielectric

Based on the characteristics of ceramics and polymers (i.e., high dielectric, low breakdown and efficiency of ceramics and high breakdown and efficiency, low dielectric constant of polymers), the preparation of ceramics and polymers into 2D–2D (layered) composite dielectrics seems to enable the two materials to complement each other and is a solution to the bottleneck problem of energy storage dielectrics. Unfortunately, there are few studies on the growth of polymers in ceramics. More attention has focused on growing wide gap inorganic such as boron nitride^[^ ^77^ ^]^ and silicon oxide^[^ ^78^ ^]^ on polymers. When designing this class of dielectrics, the main issue to be considered is the compatibility of ceramic and polymer preparation processes.

#### 3.3.1 Inorganic Barrier Layer

**Figure 37:**
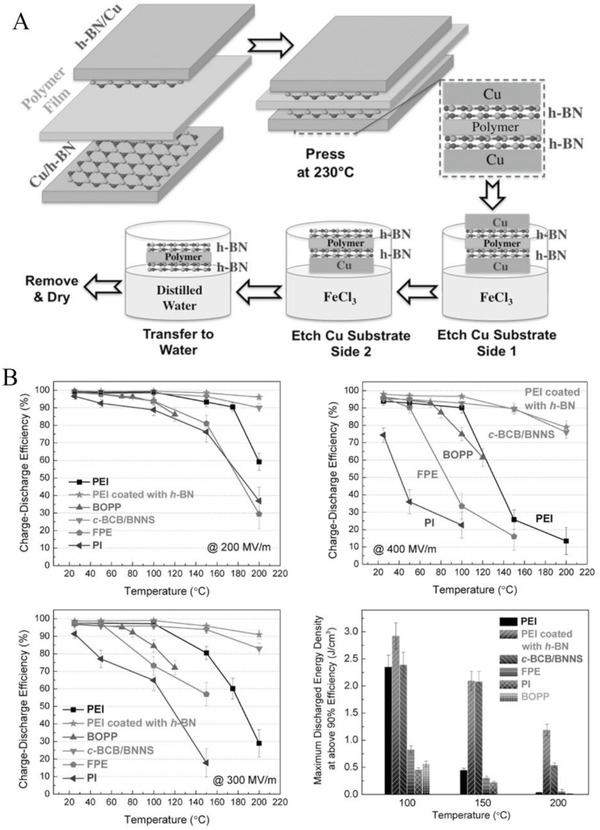
A) Schematics of the transfer process of the CVD‐grown h‐BN films onto the polymer films. B) Energy storage density of various dielectrics. Reproduced with permission.^[^ ^77^ ^]^ Copyright 2017, Wiley‐VCH.

Coating the polymer film surface with a wide band gap material provides a charge injection barrier at the interface between the electrode and the dielectric contact, which led to a prominent reduction in conductance, which is the main loss mechanism for dielectric materials working at high temperatures and in high fields.^[^ ^228^ ^]^ Hexagonal boron nitride is the structural analog of graphite, where alternating boron and nitrogen atoms replace carbon atoms in the sp2 lattice structure and result in a large band gap of ≈5.97 eV^[^ ^229^ ^]^ and has good thermal conductivity.^[^ ^230^ ^]^ The BN film obtained by chemical vapor deposition is transferred to the high temperature resistant polymer polyetherimide (PEI) and superior high‐temperature energy storage performance have been achieved. The h‐BN‐coated PEI can operate with more than 90% charge–discharge efficiencies under high electric fields even at the temperature very close to its *T*_g_ (217 °C), where pristine PEI films nearly fail. BN has also been designed to coat other polymer surfaces, such as PC.^[^ ^231^ ^]^ All achieved excellent performance, indicating the universality of the method (**Figure** **37**).

**Figure 38:**
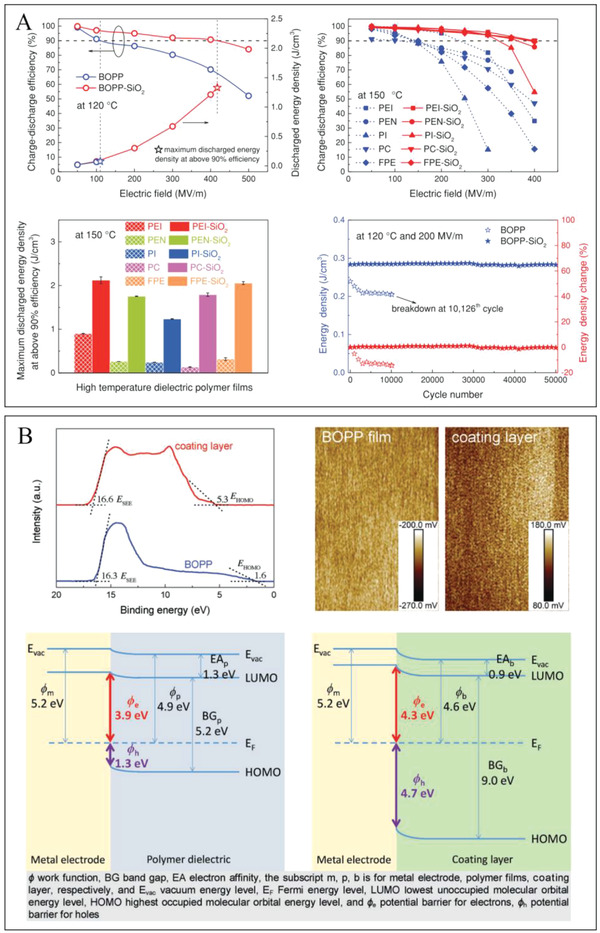
A) Energy storage characteristics, temperature stability, and charge/discharge cycles of various dielectrics before and after coating with silica. B‐a) Ultraviolet photoelectron spectra of BOPP and the SiO_2_ coating layer. b) Kelvin probe force microscopy images of the surface potential of BOPP and the SiO_2_ coating layer. c,d) Band diagrams at the electrode/dielectric interface of Au/BOPP and Au/SiO_2_. Reproduced with permission.^[^ ^78^ ^]^ Copyright 2017, Wiley ‐VCH.

Due to the low yield and complexity of the method by transferring high‐barrier inorganic films, Li et al. proposed a general and scalable approach method to modify the surface of dielectric by uniformly deposit wide‐bandgap silica (SiO_2_) onto polymer films under ambient temperature and atmospheric pressure.^[^ ^78^ ^]^ According to the experimental and simulation results, the introduced SiO_2_ layer increases the potential barrier at the electrode/dielectric interface, hinders the charge injection into the electrode, and the space charge density is greatly reduced. As a result, the high‐temperature capacitive performance of the SiO_2_‐coated dielectric is substantially enhanced compared to that of the pristine polymer. Dong et al. also studied broad‐gap oxides other than boron nitride, such as alumina, magnesium oxide, and zirconium oxide.^[^ ^232^ ^]^ The results show that alumina can significantly improve the performance of PI (**Figure** **38**).

#### 3.3.2 Inorganic Layer–Organic Layer

In addition to coating the dielectric with a broadband inorganic polymer, the introduction of an inorganic layer in the middle of the polymer can be considered to improve the energy storage properties of the dielectric. By a facile layer‐by‐layer scraping method, Huang et al. put the wide forbidden band inorganic material BNNS in the midst of the dielectric.^[^ ^233^ ^]^ The results show that this composite dielectric shows a very remarkable suppression of leakage current compared to the pristine PVDF. Computational simulations show that the compact and continuous interlayer of BNNSs can largely mitigate the local electric field distortion, thus blocking the propagation of the breakdown path. According to the experimental data, this structure provides a high breakdown strength and excellent energy storage characteristics. Wang et al. interposed 2D montmorillonite nanosheets in sandwich‐structured barium titanate/polyamide‐imide film and found that due to the anisotropy of the nanosheets, the conductive currents through the positive and negative levels were suppressed and the efficiency at 150 °C was improved.^[^ ^234^ ^]^

**Figure 39:**
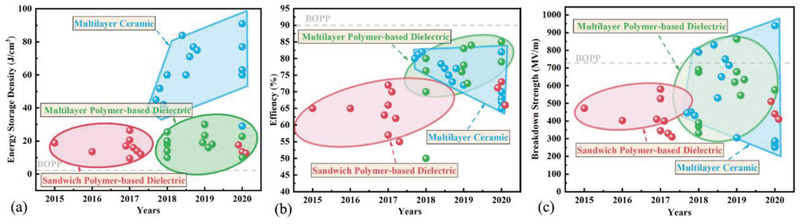
Comparison graphs of a) energy storage density, b) efficiency and c) breakdown strength for different multilayer systems.^[^ ^62^, ^91^, ^98^, ^149^, ^155^, ^171^, ^180^, ^182^, ^183^, ^185^, ^196^, ^197^, ^198^, ^199^, ^223^ ^]^

**Figure** **39**, **40** summarizes the parameters of energy storage characteristics of representative multilayer dielectrics of different systems, including multilayer ceramic dielectrics and multilayer polymer‐based composite dielectrics. Among them, sandwich and multilayer structures (more than three layers) are labeled as two separate categories due to the time of emergence of the study and the great differences in the dielectric material properties. Their performance characteristics can be clearly seen in the figure. First, the ultra‐high dielectric constant of ceramic dielectrics and the improvement of the preparation process in recent years have led to their high breakdown strength, resulting in a very high energy storage density (40–90 J cm^–3^). The energy storage density of polymer‐based multilayer dielectrics, on the other hand, is around 20 J cm^–3^. In this aspect of energy storage efficiency, the sandwich structure polymer‐based dielectric is the lowest at around 65%, followed by multilayer ceramic dielectric at around 77%, and the highest is multilayer polymer‐based dielectric at around 80%. In terms of breakdown strength, sandwich polymer‐based dielectrics are still the lowest, and multilayer ceramic dielectrics and polymer‐based dielectrics are essentially equivalent. It is worth noting that although multilayer ceramic dielectrics have better energy storage properties, they are very expensive to prepare, compared to multilayer polymer‐based dielectrics, which have very low preparation costs, and multilayer polymer‐based dielectrics may have greater commercial potential.

## 4 Preparation Methods and Manufacturing Technology for Multilayer Structure Dielectrics

### 4.1 Radio‐Frequency Magnetron Sputtering

Radio‐frequency magnetron sputtering is mainly used for the deposition of inorganic dielectric. It is used both for the preparation of stacked ceramic dielectrics such as BZT‐BST, PbTiO_3_/SrTiO_3_, PZT/PZ, BT/ST etc., and for the deposition of inorganic materials such as silica on organic substrates to prepare inorganic–organic multilayer dielectrics.

**Figure 40:**
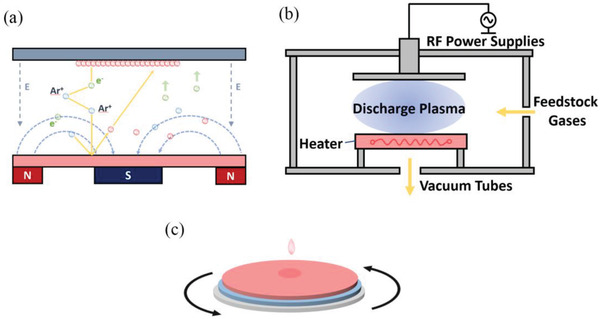
a) Schematic diagram of magnetron sputtering system. b) Schematic diagram of plasma‐enhanced chemical vapor deposition system. c) The schematic illustration for the spin‐coating method.

The sputtering phenomenon was first observed in 1852 and was considered a “dirt effect.” It was not until more than a century later that the process began to be utilized by industry. The simplest layout of sputtering equipment is a DC‐powered diode, i.e., a low‐voltage glow discharge is ignited in the space between two planar electrodes. The substrate to be coated is the anode and the cathode is the target, which is corroded due to bombardment by high‐energy argon ions generated in the gas discharge. The atoms move toward the substrate, where a thin film is formed. For simultaneous sputtering of highly insulating target materials (e.g., SiO_2_ or Al_2_O_3_), a plasma discharge driven by radio frequency with an operating frequency of 13.56 MHz is introduced.^[^ ^235^ ^]^

During magnetron sputtering, the material on the target is transferred to the target substrate, where no chemical but only physical changes take place. By changing the target material periodically during the deposition process, ceramic dielectrics with a layered structure can be obtained. The thickness of a single layer can be regulated by controlling the time and rate of magnetron sputtering of each layer.^[^ ^98^, ^236^ ^]^ But the method also has significant limitations when it comes to preparing organic‐inorganic multilayer dielectric. Inorganic materials with a chalcogenide structure generally have higher crystallization temperatures than those tolerated by organic materials, so the choice of material is limited in many ways when depositing inorganic layer.

### 4.2 Plasma Enhanced Chemical Vapor Deposition

Chemical vapor deposition is a process technology in which reacting substances under gaseous conditions undergo a chemical reaction to produce a solid substance deposited on the surface of a heated solid substrate, resulting in a solid material. Plasma enhanced chemical vapor deposition is a form of chemical vapor deposition. This method can be used to prepare highly insulating compounds such as silica and boron nitride on organic surfaces.^[^ ^77^, ^237^ ^]^

It should be noted that due to the high reaction temperature of boron nitride, inorganic–organic composite films can be prepared by preparing a boron nitride film on a metal substrate, preparing an organic film on this film and later etching away the metal. In addition, Li et al. have improved the PECVD by using a carefully controlled microsecond‐pulse voltage with optimized ramping rate, frequency, and amplitude.^[^ ^78^ ^]^ After this improvement, the output of the process of depositing silica on the surface of the polymer film has been greatly improved, which is very conducive to the integration of actual production.

### 4.3 Spin Coating

The spin‐coating method is mainly used to prepare multilayer inorganic ceramic dielectric. Spin‐coating method involves spin‐coating the precursor solution onto the substrate and obtaining a stable and dry film after heat treatment.^[^ ^10^, ^238^, ^239^ ^]^ Multilayer structural ceramics are obtained by alternate growth. The thickness of each monolayer can be accurately manipulated by tuning the deposition parameters, which include the viscosity and concentration of the precursor solution as well as the speed and time of the spin coating process. Different numbers of stacking cycles can be achieved by adjusting the deposition sequence of different precursors.

The advantage of this method is that it is simple and fast, and the disadvantage is that the product quality is not high. Compared with other preparation methods, this method is very simple, with high preparation volume and low cost. However, the thickness of the monolayer is difficult to be reduced to the nanometer level due to the low control accuracy. Moreover, the quality of the membranes is relatively poor.

### 4.4 Coextrusion

Multilayer coextrusion is an advanced polymer processing technology that combines multiple polymers in a controlled structural layered structure. It is mainly used to prepare multilayer all‐organic composite dielectrics. It is a continuous processing technology capable of producing multilayer films with a wide range of monolayer thicknesses (from microns to nanometers). It is also very low cost and has great commercial value.^[^ ^240^ ^]^

**Figure 41:**
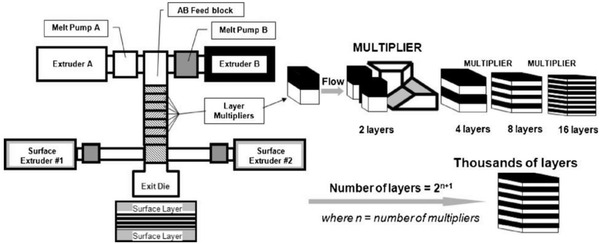
Two‐component multilayer system and diagram of layer multiplication through cutting, expanding, and reorganizing the melt flow.^[^ ^219^ ^]^

Specifically, a two‐component co‐extrusion system is shown in **Figure** **41**. The system consists of two single‐screw extruders with melt metering pumps, a two‐layer co‐extrusion feed block, a series of laminated die head elements, two laminated surface extruders, and an outfeed strip or film die head. In particular, a metering pump is used to control the relative thickness ratio of the layers of polymer entering the feed zone. Two layers of polymer enter the feed zone into a series of laminating dies, each doubling the number of layers by a process of cutting, spreading, and stacking the viscoelastic melt. The series of n doubling elements combine the two polymers into alternating layers to 2*^n^* ^+1^. The thickness of each layer can be controlled by adjusting the speed of the rotor. However, this method is limited to materials with good flowability.

### 4.5 Electrospinning Technology

Electrostatic spinning technology is mainly used to prepare multilayer polymer‐based dielectrics. In addition to the preparation of 1D nanofiber fillers, electrostatic spinning techniques can be used in the preparation of multilayer nanocomposite dielectrics or heterogeneous structured multilayer dielectrics. In the preparation of multilayer energy storage dielectric using electrostatic spinning technology, there are often two methods: one is to electrospin multiple single‐layer dielectric films separately, and then hot‐press them into one multilayer film in a certain order; the other is to form multilayer dielectric directly on the receiver by changing the precursors in a layer‐by‐layer spinning way, and then hot‐press them to obtain a denser dielectric.^[^ ^192^ ^]^

**Figure 42:**
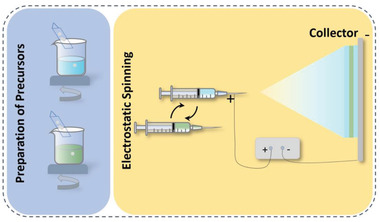
Schematic diagram of the electrostatic spinning process.

As shown in **Figure** **42**, a typical electrospinning device is mainly composed of three basic elements: a high‐voltage power supply, a spinneret, and a grounded collector. The two electrodes of the high‐voltage power supply are respectively connected to the spinneret and the collector containing the spinning solution. When electrostatic spinning is performed, the precursor solution is extruded from the spinnerette head in the presence of an electric field to form small droplets, and then the charged solution spinnerette is extruded from the cone. Typically, fluid elongation occurs first in uniform filaments, and then the linear streamlines undergo violent churning and/or splitting motions due to fluid and electrodynamic bending instabilities.^[^ ^241^ ^]^ Finally, continuous primary spun fibers are usually deposited on the collector in the form of a two‐dimensional nonwoven fiber web. The fiber morphology can be controlled by controlling some parameters. For example, the concentration of precursors, electrical conductivity, and surface tension.^[^ ^242^ ^]^

Electrostatic spinning technology can adjust the structure of inorganic‐organic multilayer dielectrics in a wide range, but the yield is low.

## 5 Challenges and Perspectives

In this review, we systematically summarize the recent advances in ceramic energy storage dielectrics and polymer‐based energy storage dielectrics with multilayer structures and the corresponding theories, including interfacial polarization, electric field distribution characteristics of multilayer dielectric species, and breakdown hindrance effects. Exciting advances have been made in recent years. Multilayer structures are useful for enhancing the energy storage properties of dielectrics. The design ideas of multilayer structured dielectrics are sorted out. The energy storage characteristics of different kinds and structures of multilayer dielectrics are summarized.

However, through the combing, what can be found at the same time is that there are some theories that are still unclear and there are some phenomena whose causes are still not unanimously agreed. It is very obvious that deeper mechanism‐related studies deserve to be given efforts.

1) First, most of the current partial pressure models in multilayer dielectric are applicable to multilayer composite systems with a constant dielectric constant, i.e., linear–linear dielectric composite system. For most inorganic multilayer energy storage dielectrics and organic multilayer energy storage dielectrics composed of PVDF, the constituent units are often ferroelectric or antiferroelectric materials. The dielectric constant of ferroelectric/antiferroelectric materials varies with the applied electric field and their polarization characteristics are complex. This results in a dynamic process in which the voltage assigned to each layer of a multilayer dielectric varies as the applied electric field is gradually increased. This process can be obtained by a preliminary analysis of the polarization characteristics of single‐layer dielectrics. The current research in this area is still relatively weak, and it is possible to obtain a multiseries system with high energy storage density and high energy storage efficiency by matching the polarization curves of a variety of linear, ferroelectric and antiferroelectric dielectric materials. Sound research in this area will help to design the structural composition of multilayer composite dielectrics from the source.
2) The effect of the direction perpendicular to the electric field on the dielectric breakdown path has been largely limited to simulations, with few experimental results demonstrating the existence of this effect. Although this effect was discovered at a very early stage, the dielectric material for capacitors is unique and different from the previous experimental study objects and conditions (e.g., film size, applied voltage waveform), so relevant experimental studies are still indispensable. If experimental studies in this area can be improved to fill the theoretical and research gaps related to multilayer dielectrics.
3) It should be noted that the microscopic characteristics of the layer interface in different multilayer systems are different, for example, the microscopic composition and phase structure of the layer and layer in inorganic multilayer dielectric are often different, and the interface is a real interface at this time. For the multilayer dielectric composed of the same matrix, by filling with different content of inorganic filler, whether there is a real “interface” between the layer and the layer is subject to further analysis, because the organic matrix material is the same, only because of the existence of filler and the redistribution of the electric field distribution in the multilayer dielectric. For the above interfaces, the study of the relationship between the interface and the electrical properties needs to be treated differently.
4) In addition, studies on the influence of the interface on the polarization characteristics are relatively lacking. Since a large amount of interfacial polarization caused by interfaces may exist in multilayer systems, the study of such polarization characteristics is important for understanding the energy storage behavior and guiding the performance tuning of multilayer dielectrics. In particular, interfacial polarization in different materials has a large response time difference, and the response of interfacial polarization varies in different application scenarios in conjunction with the applied voltage waveform, which cannot be generalized. However, this problem has not yet attracted sufficient attention.
5) It is well known that inorganic ceramic dielectrics usually have a high dielectric constant and low breakdown strength, while organic dielectrics have a high breakdown strength and a low dielectric constant. Therefore, the combination of the two can theoretically be effective in increasing the breakdown strength and dielectric constant. However, the preparation processes of organic and inorganic materials are usually incompatible. For example, the crystallization process of inorganic materials usually requires high‐temperature treatment, but organic materials cannot withstand high temperatures, and organic materials often need to be treated by stretching, but the rigidity of inorganic dielectrics means that they cannot withstand this process. How to prepare organic–inorganic composite dielectrics through existing processes or develop new ones, so that the respective performance advantages of organic and inorganic dielectrics can be exploited and ultimately high energy storage density and efficiency can be obtained is the focus of attention for researchers. Simple, low‐cost techniques for the preparation of multilayer dielectrics are to be further developed.

Despite these problems, multilayer dielectric as the most favored energy storage dielectric, there is still room for performance improvement and theoretical development. The following are perspectives for multilayer energy storage dielectrics.

As introduced in Section 2.2.1, the introduction of the nonlinear *P‐E* curves based on the partial electric field equation means that it is possible to predict the energy storage density and energy storage efficiency of double‐layer or multilayer dielectric based on the *P‐E* curves of the single‐layer dielectrics. In addition, *P‐E* curves for a variety of monolayer dielectrics have been reported in the literature and can be used as initial data for selecting multilayer dielectric composition materials. The selection of dielectric material is based on the results of the calculations, which will shorten the time for preliminary material selection. In addition, building a database of the *P‐E* curves of the dielectric by high‐throughput calculations, machine learning or deep learning is also a novel and effective way to predict the performance of multilayer energy storage dielectrics.

In addition to the performance coupling relationship between the two dielectrics, the most important thing in multilayer dielectric is the interface effect. For energy storage properties, the main concern is the effect of different interfaces on breakdown and polarization. Regarding the interface and breakdown, the relationship between structural parameters and breakdown performance can be obtained by designing different interfacial microstructures. For inorganic multilayer dielectrics, the preparation process of one layer on another layer is regulated so as to obtain interfacial regions with different lattice matching degree, crystallization degree, and other microscopic properties. The conformational relationship between process‐microstructure‐property can be obtained after testing, and then by regulating the number of layers, the relationship between the number of layers and breakdown can be obtained, so as to finally determine the selected interface properties and the number of interfaces in multilayer dielectric; For organic multilayer dielectric, the regulation of the interface is mainly reflected in the regulation of the hot pressing process or extrusion process, and the preparation process is different from inorganic while the research idea is similar to inorganic.

As mentioned above, the problem of preparing organic–inorganic composite multilayer dielectric is the incompatibility of the preparation process between the two materials. Organic materials usually cannot be prepared at temperatures higher than 300 °C, however, inorganic materials are often prepared at temperatures higher than it. Therefore, it is crucial to find methods that allow the preparation of inorganic layers at low temperatures. PECVD is one of the most promising methods according to the current research.

## Conflict of Interest

The authors declare no conflict of interest.
